# Cell Wall Compositions of *Sorghum bicolor* Leaves and Roots Remain Relatively Constant Under Drought Conditions

**DOI:** 10.3389/fpls.2021.747225

**Published:** 2021-11-12

**Authors:** Tess Scavuzzo-Duggan, Nelle Varoquaux, Mary Madera, John P. Vogel, Jeffery Dahlberg, Robert Hutmacher, Michael Belcher, Jasmine Ortega, Devin Coleman-Derr, Peggy Lemaux, Elizabeth Purdom, Henrik V. Scheller

**Affiliations:** ^1^Department of Plant & Microbial Biology, University of California, Berkeley, Berkeley, CA, United States; ^2^Joint BioEnergy Institute, Emeryville, CA, United States; ^3^Environmental Genomics and Systems Biology Division, Lawrence Berkeley National Laboratory, Berkeley, CA, United States; ^4^Department of Statistics, University of California, Berkeley, Berkeley, CA, United States; ^5^Berkeley Institute for Data Science, University of California, Berkeley, Berkeley, CA, United States; ^6^DOE Joint Genome Institute, Berkeley, CA, United States; ^7^Kearney Agricultural Research and Extension Center, University of California, Parlier, Parlier, CA, United States; ^8^West Side Research and Extension Center, University of California, Five Points, Five Points, CA, United States; ^9^Plant Gene Expression Center, United States Department of Agriculture-Agricultural Research Service, Albany, CA, United States

**Keywords:** *Sorghum bicolor*, drought, cell wall, biomass conversion and expansion factor (BCEF), pre-flowering, post-flowering

## Abstract

Renewable fuels are needed to replace fossil fuels in the immediate future. Lignocellulosic bioenergy crops provide a renewable alternative that sequesters atmospheric carbon. To prevent displacement of food crops, it would be advantageous to grow biofuel crops on marginal lands. These lands will likely face more frequent and extreme drought conditions than conventional agricultural land, so it is crucial to see how proposed bioenergy crops fare under these conditions and how that may affect lignocellulosic biomass composition and saccharification properties. We found that while drought impacts the plant cell wall of *Sorghum bicolor* differently according to tissue and timing of drought induction, drought-induced cell wall compositional modifications are relatively minor and produce no negative effect on biomass conversion. This contrasts with the cell wall-related transcriptome, which had a varied range of highly variable genes (HVGs) within four cell wall-related GO categories, depending on the tissues surveyed and time of drought induction. Further, many HVGs had expression changes in which putative impacts were not seen in the physical cell wall or which were in opposition to their putative impacts. Interestingly, most pre-flowering drought-induced cell wall changes occurred in the leaf, with matrix and lignin compositional changes that did not persist after recovery from drought. Most measurable physical post-flowering cell wall changes occurred in the root, affecting mainly polysaccharide composition and cross-linking. This study couples transcriptomics to cell wall chemical analyses of a C4 grass experiencing progressive and differing drought stresses in the field. As such, we can analyze the cell wall-specific response to agriculturally relevant drought stresses on the transcriptomic level and see whether those changes translate to compositional or biomass conversion differences. Our results bolster the conclusion that drought stress does not substantially affect the cell wall composition of specific aerial and subterranean biomass nor impede enzymatic hydrolysis of leaf biomass, a positive result for biorefinery processes. Coupled with previously reported results on the root microbiome and rhizosphere and whole transcriptome analyses of this study, we can formulate and test hypotheses on individual gene candidates’ function in mediating drought stress in the grass cell wall, as demonstrated in sorghum.

## Introduction

As fossil fuel resources decline and usage continues to have adverse effects on global climate, it is crucial to develop alternative fuel sources to supply energy demand. Lignocellulosic bioenergy crops have the advantage of being renewable energy resources with reduced carbon emissions compared to petroleum-based fuel. Recent studies have demonstrated that the use of lignocellulosic biofuels has the potential to sequester carbon from the atmosphere by increasing soil organic carbon, which may help mitigate increased carbon release to the atmosphere (Vanholme et al., 2013; Taptich et al., 2018; Wu et al., 2018). Despite these advantages, lignocellulosic biofuels have several requirements to meet before they can be considered competitive with petroleum-based fuels. The foremost requirement is cost competitiveness, which is primarily dependent on the recalcitrance of the cell wall and the ability to valorize waste products from biofuel production. Cell wall recalcitrance can often be simplified to several key factors: the amount and complexity of lignin, the amount and crystallinity of cellulose, the presence of biomass conversion inhibitors such as acetate, and the ratio of C5:C6 sugars, with a low ratio tending to favor saccharification efficiency and conversion into fuels (Somerville et al., 2010; Bosch and Hazen, 2013; van der Weijde et al., 2013; Kumar and Sharma, 2017; Kim, 2018). In addition to biomass conversion efficiency, lignocellulosic feedstocks should ideally be grown on land that is not suitable for food crops to avoid displacing food crops. A major factor that makes cropland marginal is low rainfall and/or increased drought. With irrigation water being a severely limited, expensive, and energetically intensive resource, lignocellulosic feedstocks must be robust enough to generate high biomass yields even under adverse conditions like drought. In addition to high biomass yields, the lignocellulosic properties of the feedstocks would ideally not be altered by drought (Somerville et al., 2010; van der Weijde et al., 2013; Vanholme et al., 2013).

The impact of drought on the plant cell wall-related genes has been investigated in transcriptomic and proteomic studies (Wu et al., 2001; Vincent et al., 2005; Zhu et al., 2007; Sasidharan et al., 2011; Le Gall et al., 2015; Nakano et al., 2015; Tenhaken, 2015; Lenk et al., 2019). Over the last several years, researchers have also performed basic chemical analyses of plant cell walls produced under drought conditions. However, these studies are limited in several ways. First, most transcriptomic studies lacked cell wall analysis to verify that transcriptomic changes translated into chemical changes in the wall. Secondly, much of the chemical cell wall analyses that were performed were either on specialized tissue types, from multiple plant lineages, or were under conditions unlikely to be encountered outside of a laboratory (Iraki et al., 1989; Wakabayashi et al., 1997; Piro et al., 2003; Konno et al., 2008; Leucci et al., 2008; Rakszegi et al., 2013). Thirdly, more recent studies analyzing compositional and sugar yield differences use techniques that do not distinguish between different polysaccharides nor are they coupled to transcriptomic analyses (Emerson et al., 2014; Ottaiano et al., 2017; van der Weijde et al., 2017; Hoover et al., 2018). These variables can make it incredibly difficult to draw anything other than very broad conclusions for a given plant on how the cell wall will respond to drought.

Several grasses have been proposed as lignocellulosic feedstocks including: *Sorghum bicolor, Panicum virgatum*, and *Miscanthus* spp. (van der Weijde et al., 2013; Mullet et al., 2014). These grasses have all been selected for their high vegetative biomass yields and for their increased tolerance to prolonged drought and nutrient scarcity (van der Weijde et al., 2013; Mullet et al., 2014). *S. bicolor* has several advantages as a crop and as an experimental system including: high yield under water and nitrogen-deficient conditions, a sequenced genome, genetic tractability, and demonstrated genetic modification techniques (Howe et al., 2006; Liu and Godwin, 2012; van der Weijde et al., 2013; Mullet et al., 2014; Wu et al., 2014; Abdel-Ghany et al., 2016; McCormick et al., 2018). Furthermore, *S. bicolor* has the advantage of having different either pre-flowering drought-tolerant or post-flowering drought-tolerant cultivars (Xu et al., 2000; Borrell et al., 2014). For this study, we used RTx430, a pre-flowering drought-tolerant line, and BTx642, a post-flowering drought-tolerant line (Xu et al., 2000; Borrell et al., 2014), to explore how some different drought-tolerance strategies affect cell wall composition under both pre-flowering and post-flowering drought stress.

## Materials and Methods

### Sorghum Growth Conditions and Drought Induction

This work was done in collaboration with the DOE BER-funded project EPICON (Epigenetic Control of Drought Response in *S. bicolor*; Grant DE-SC0014081) and utilized the same plant material that has previously been published (Xu et al., 2018; Varoquaux et al., 2019). Sorghum planting, field conditions, and irrigation can all be found here (Xu et al., 2018; Varoquaux et al., 2019). Briefly, sorghum plants were grown in Parlier, CA, United States (36.6008°N, 119.5109°W) for 17 weeks, with week 0 starting on June 01 with seedling emergence. Plants were subjected to three different growth conditions: an irrigated control condition with weekly drip irrigation consisting of 80% calculated evapotranspiration 5 days prior to the weekly sampling date for the duration of the experiment, a pre-flowering drought condition with no watering (weeks 3–8) followed by weekly watering and recovery during post-anthesis, and a post-flowering drought condition with no water (weeks 10–17) during post-anthesis. Eighteen plots of randomly assigned irrigation conditions and genotypes were planted with three replicates per condition per genotype. The third and fourth fully emerged leaf from the primary tiller was collected each week from ten plants per plot and pooled into a single sample for each plot resulting in three pooled samples for each genotype for each condition, bagged in foil and flash-frozen in liquid nitrogen. Additionally, the roots of ten plants per plot (about 30 cm in depth from topsoil) were also collected each week and pooled into a single sample as described for the leaves, vortexed for 2 min in epiphyte removal buffer (0.633% NaH_2_PO_4_⋅H_2_O, 1.65% NaH_2_PO_4_⋅7H20, 200 μl Silwet-77/L), washed twice in root washing buffer (0.633% NaH_2_PO_4_⋅H_2_O, 1.65% NaH_2_PO_4_⋅7H_2_0), gently dried, bagged in foil, and flash-frozen in liquid nitrogen. All tissues were collected the same day of the week at the same time of day (10 am to 1 pm). We studied two *S. bicolor* lines; the pre-flowering “early senescing” drought-tolerant RTx430 and the post-flowering “stay green” drought-tolerant BTx642 to better understand how lines that may fall under either drought tolerance strategy fare.

### Computational Methods

We downloaded transcriptomic data and preliminary statistical analysis from samples obtained from the same field experiment. The transcriptomic data consists of almost 400 samples of leaf and root tissues under well-watered conditions, pre- and post-flowering drought stress, and a pre-flowering drought recovery period, for both RTx430 and BTx642.

The full computational pipeline is described in Varoquaux et al. (2019). Briefly, raw data was checked for quality analysis and one sample of low-quality was removed, resulting in 198 samples for leaves and 198 samples for roots. We then filtered low expressed genes and applied upper-quartile normalization on the remaining set of genes using EDA-seq (Bullard et al., 2010; Risso et al., 2011). Differential expression analysis was performed using a method akin to EDGE (Storey et al., 2005): we modeled the gene expression data as a smooth function over time, with a different functional form estimated for each watering condition and genotype, and identified genes differentially expressed between the control condition and the two drought conditions over time for both genotypes. We also identified genes differentially expressed between the two genotypes. The data and results of these analyses are available on https://www.stat.berkeley.edu/~epicon/publications/rnaseq/.

### Identifying “Highly-Variable” Genes

We then identified highly variable genes (HVGs) for leaf and root samples as follows. First, for each sample and drought condition, we selected genes differentially expressed across both genotypes by combining *p*-values obtained as described above using Fisher’s method (Yates and Fisher, 1971). We thus obtained for each gene, each drought condition, and each sample type a unique p-value assessing whether this gene is affected by drought. We then corrected for multiple tests using Benjamini-Hochberg. Second, we computed the overall log-fold change across the time-course experiment using the method described in Varoquaux et al. (2019) by applying the following formula:


$${\text{lfc}}_{i}^{C}=\text{sign}\,\left(\frac{1}{T}\,\sum_{t=1}^{T}L_{i}^{C,t}\right)\times \left(\frac{1}{T}\,\sum_{t=1}^{T}L_{i}^{C,t}\right),$$

where $L_{i}^{C,t}$ corresponds to the log-fold change of gene *i* at time *t* and for each drought condition *C*. The vector $L_{i}^{C}\in {\mathbb{R}}^{T}$ is estimated by limma (Law et al., 2014).

For each drought condition and each genotype, we ranked genes found differentially expressed (adjusted *p*-value < 0.05) and labeled the top 5% and bottom 5% as highly variable, obtaining four distinct lists (RTx430 – Pre-flowering drought, RTx430 – Post-flowering, BTx642 – Pre-flowering, and BTx642 – Post-flowering). We then applied a similar procedure on Pre-flowering conditions, considering only time-points during drought and time-points during recovery, thus obtaining an additional four lists. We then merged the 8 lists of “highly-variable” genes to obtain a set of genes highly affected by drought in leaves and in roots.

Highly variable genes were categorized via assigned gene ontology (GO) terms related to cell wall-specific components or processes outlined in Table 1 and were analyzed further, comparing relative expression levels at weeks 7 and 14 (Supplementary File 1). Expression analysis and tissue analysis focused on weeks 7 and 14 to represent pre-flowering and post-flowering time points, respectively. An additional set of hand-selected putative cell wall-related HVGs were also compiled based on preliminary analysis of leaf and root tissues at week 13 (data not shown). All putative glycosyltransferases and glycosyl hydrolases (GH) were referenced against a recent phylogenetic analysis of *S. bicolor* Carbohydrate Active enZyme (CAZy) families (Rai et al., 2016). Cell wall-related HVGs were split into biosynthesis, modification, and signaling categories and main trends in differential expression between the three different conditions when compared to well-watered plants were assigned (increased, decreased, or no change in expression during the bulk of the condition).

**TABLE 1:** Sources of cell-wall related highly variable genes (HVGs) analyzed in this study.

Source	GO term	GO number
Curated list – based on preliminary chemical analysis of RTx430 from week 14	N/A	N/A
GO association	Plant-type cell wall biogenesis	0009832
GO association	Cell wall organization	0071555
GO association	Cell wall organization or biogenesis	0071554
GO association	Cell wall biogenesis	0042546

### Cell Wall Extraction and Isolation

The alcohol insoluble residue (AIR) was extracted from frozen, ground plant tissue as described by Harholt et al. (2006) with modifications. Briefly, samples were boiled and successively washed in 100% ethanol until no pigment remained. Pellets were then washed in 70% ethanol followed by 100% acetone and dried in a 50°C oven overnight. AIR extracts were then subjected to starch removal as described by Harholt et al. (2006) with modifications. AIR samples were subjected to a two-step enzymatic degradation with an initial 10 min thermostable α-amylase (Megazyme, E-BLAAM) digestion at 85°C followed by a 2-h amyloglucosidase (Megazyme, E-AMGDF) and pullulanase (Megazyme, E-PULBL) digestion at 50°C. Samples were washed twice with 70% ethanol after enzymatic digestion before drying the pellet via a solvent concentrator.

### Cell Wall Hydrolysis

The hemicellulosic and pectic matrix of 0.5–2 mg de-starched AIR samples were hydrolyzed in 1 ml 2 M trifluoroacetic acid at 120°C for 1 h. Trifluoroacetic acid (TFA) was removed via a solvent concentrator and the pellet was resuspended in 1 ml of nanopure water. Samples were then filtered through a 0.45 μm centrifugal filter and taken for analysis of released matrix monosaccharides.

The pellets of TFA-hydrolyzed AIR samples were washed twice in isopropanol to remove all hydrolyzed sugars and leave the cellulosic components of the AIR samples. Cellulosic sugars were released by Saeman’s hydrolysis (Saeman, 1945) by incubating in 63 μl of 72% (v/v) sulfuric acid at room temperature for 1 h and subsequently diluting the samples in water to a final concentration of 1 M sulfuric acid and incubating for an additional 3 h at 100°C. Sulfuric acid was neutralized and precipitated using solid barium carbonate and removed by centrifugation. Samples were dried via a solvent concentrator before re-suspending in 250 μl of nanopure water for cellulosic monosaccharide composition analysis.

### Monosaccharide Composition Analysis

Matrix and cellulosic acid-hydrolyzed AIR samples were detected and quantified using High Performance Anion Exchange Chromatography with Pulsed-Amperometric Detection (HPAEC-PAD) as described by Voiniciuc and Gunl (2016) with some modifications using a Thermo Scientific Dionex ICS-5000 system. Neutral sugars were separated over a 4 – 1 mM sodium hydroxide 0.4 ml/min gradient over 23 min before separating the uronic acids using 450 mM sodium hydroxide at 0.4 ml/min over 18 min using a Dionex CarboPac PA20 column (3 mm × 30 mm, 060144). Amounts were quantified using a range of monosaccharide standards (2.5–200 μM).

### Ferulic Acid Content Determination

Ferulic acid was extracted from de-starched AIR samples as described by Eudes et al. (2012). Briefly, hydroxycinnamates were extracted from 5 to 10 mg de-starched AIR samples in 2 M sodium hydroxide at 30°C overnight with light shaking (300 rpm) before acidification with hydrochloric acid. Ferulic acid and other hydroxycinnamates were isolated via three successive ethyl acetate extractions. Extracted hydroxycinnamates were dried and resuspended in 50% methanol before quantification via High Performance Liquid Chromatography with Diode Array Detection (HPLC-DAD) using the Agilent 1200 Series system. Hydroxycinnamates were eluted on a 10 mM ammonium acetate flow with a gradient acetonitrile mobile phase over 15.6 min using the Agilent Eclipse Plus Phenyl-Hexyl column (4.6 mm × 250 mm, 959990-912). Acetonitrile mobile phase gradients were 27% from 0–12 min, 72% from 12–12.1 min, 90% from 12.1–12.8 min, and 27% from 12.8–15.6 min.

### Lignin Quantification

Soluble lignin content was determined and calculated using the acetyl bromide method as described by Barnes and Anderson (2017). De-starched AIR samples (5 mg) were hydrolyzed in 25% (v/v) acetyl bromide in glacial acetic acid in a 50°C water bath for 3 h with occasional mixing. Samples were then treated with 25 mM hydroxylamine hydrochloride to produce a change in optical density and their absorbance was subsequently measured using a quartz cuvette at 280 nm. Lignin content was quantified as described by Barnes and Anderson (2017) using an extinction coefficient average (18.19509) of known commelinid extinction coefficients via (Fukushima and Hatfield, 2004).

### Saccharification Efficiency Assay

Saccharification efficiency was determined on de-starched AIR samples and AIR samples with intact starch. A hot water pre-treatment was administered by autoclaving 2 – 10 mg of AIR samples in 340 μl water at 121°C for 1 h. A 0.01% (v/v) Cellic CTec3 cellulase cocktail (Novozymes, Bagsvaerd, Denmark) in 50 mM citrate buffer pH 5.0 (650 μl) was added to each sample and incubated shaking at 800 rpm at 50°C for 72 h. Sugar content was assayed prior to incubation (T0) and every subsequent 24 h for 72 h using a 3,5-dinitrosalicylic acid colorimetric assay (Miller, 1959). Supernatant from each sample was incubated with 3,5-dinitrosalicylic acid at 95°C for 10 min. Absorbance from each sample was measured at 540 nm and samples were quantified against a linear range of glucose standards (0–2 mg).

### Statistics

Statistics of all chemical cell wall analyses were performed using the Student’s *t*-Test comparing the droughted treatment to its watered control for the specified week sampled. The resulting *p-*values were corrected for multiple comparisons concerning False Discovery Rate (FDR) using the Benjamini–Hochberg correction (Benjamini and Hochberg, 1995) to determine the statistical significance of *p*-values < 0.05. FDRs were assumed to be the conservative 0.25, and all *p* and Benjamini–Hochberg values can be found in Supplementary File 2. Lines were compared within genotypes and within tissue types to see differences pertaining to genotypes and tissue types.

## Results

### Transcriptome and Cell Wall-Related Highly Variable Genes

The previous studies of the plants used in this study showed that the applied drought stress treatments had substantial impact on plant physiology, e.g., as measured by plant height, biomass, Crop Water Stress Index (CWSI), and expression of marker genes for drought stress (Varoquaux et al., 2019). For example, CWSI, which ranges between 0 (unstressed) and 1 (100% decrease in estimated transpiration) was 0.0 in control plants at week 8, and 0.56 in preflowering drought stressed plants. At week 14 the values were 0.06, 0.11, and 0.78 in control plants, preflowering stressed (rewatered) plants, and postflowering stressed plants, respectively. In addition, transcriptomic analysis of leaf and root tissues of those plants showed a massive transcriptional response to drought stress, with a cumulative total of more than 10,000 genes affected, many of which exhibited changes in the first week of drought exposure (Varoquaux et al., 2019). Amongst the genes exhibiting a striking response to drought, many are genes known to be involved in response to abiotic stress, with putative functions including heat shock genes, response to abscisic acid, response to oxidative stress, etc. (Varoquaux et al., 2019). In particular, transcripts from two dehydrin genes (DS1 and DS6), which have been demonstrated to be molecular markers of drought stress in sorghum, showed significant overexpression (DS1: average log2-fold change = 12.7; DS6: average log2-fold change = 10.5) in preflowering stressed roots compared to control roots at 8 weeks. Similar increases in expression for both genes were observed during the post-flowering drought treatment (week 14), (DS1: average log2-fold change = 5.2; DS6: average log2-fold change = 5.1). Details are available in Xu et al. (2018).

**FIGURE 1:**
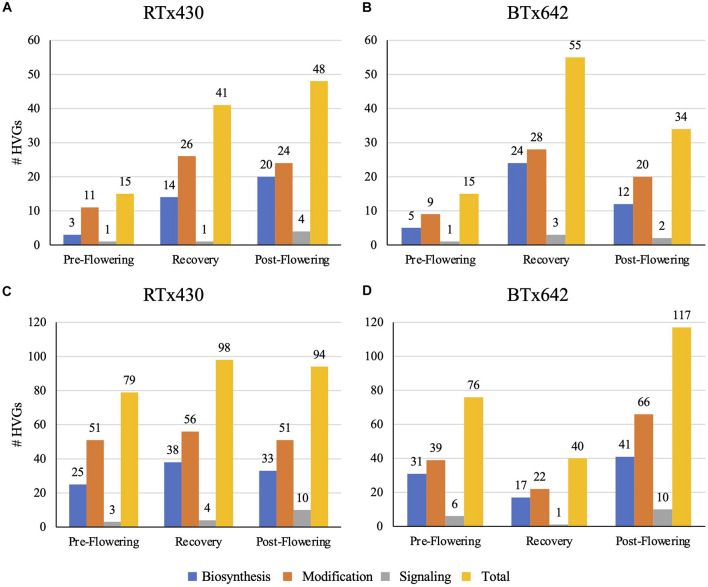
Total number of highly variable genes (HVGs) with cell wall-related gene ontology (GO) terms in leaves **(A,B)** and roots **(C,D)**. Highly variable genes are those genes in the top 10% of differentially expressed genes in response to drought conditions, of the HVGs, those genes with cell wall-related GO terms were analyzed and classed via putative functions related to biosynthesis, modification, and signaling of the cell wall.

Drought stress had different impacts on the transcriptome relating to cell wall-specific genes depending on the tissue type and the developmental time of drought induction (details on how we defined cell wall-specific genes are described in Section “Materials and Methods”). The cell wall-specific transcriptome was modified under all conditions relative to the well-watered control: pre-flowering drought induction, recovery after pre-flowering drought induction, and post-flowering drought induction. Within the cell wall-related transcriptome, we focused our analysis on HVGs, those genes in the top 10% of differentially expressed genes when referenced against the watered control ranked by log-fold change. Leaves had fewer cell wall HVGs, with 15–55 highly variable cell wall-related genes across treatments (Figure 1). Notably, leaves experiencing a pre-flowering drought from both genotypes had only 15 highly variable cell wall-related genes. Of these genes, most appeared to encode cell wall modifying enzymes such as expansins, xyloglucan endotransglucosylase/hydrolases (XTHs), peroxidases, and pectin-modifying enzymes (Figure 1 and Supplementary Figure 1). In contrast, roots experienced a greater number of cell wall-related HVG, with 40–117 highly variable cell wall-related genes across treatments (Figure 1) in which HVGs with putative modification functions also predominated (Supplementary Figure 2). A recent estimate of genes encoding enzymes related to cell wall biosynthesis and modification in sorghum is 520, largely comprised of 160 glycosyltransferases, 201 GHs, and 83 expansins (Rai et al., 2016). However, this estimate only included some well-characterized gene families, and in particular, the number of cell wall-related glycosyltransferases is certainly much higher. In fact, a broader survey of cell wall-related genes in sorghum found approximately 1,200 genes related to the cell wall, including substrate synthesis, membrane trafficking, post-depositional modification, and signaling in addition to polysaccharide biosynthesis (Carpita and McCann, 2008).

Generally speaking, the root cell wall-related transcriptome followed broad patterns across treatments. Both pre-flowering and post-flowering drought induction across genotypes resulted in a decrease in highly variable cell wall-related gene expression (Supplementary Figure 3). However, the *S. bicolor* RTx430 genotype experiencing a pre-flowering drought induction had an increase in highly variable cell wall-related gene expression upon re-watering; this effect was not seen as dramatically in the BTx642 cultivar (Supplementary Figure 3). Effected HVG predominantly encoded putative cell wall-modifying and biosynthetic enzymes, with a decrease in expression of glycosyltransferases, acetyl- and methyltransferases (Supplementary Figure 4), peroxidases, pectin modifiers (esterases, lyases, etc.), GHs, and expansins and XTHs (Supplementary Figure 5). Based on these results, we hypothesized that drought induction resulted in the decrease in cell wall deposition and modification in basal roots, with altered polysaccharide-lignin cross-linking and potentially altered pectin structure. It is interesting that pectin-related genes were so highly represented in tissues that should be predominantly secondary cell walls in a species with reportedly low pectin content (Vogel, 2008). We also hypothesize that re-watering after a pre-flowering drought induction results in increased cell wall deposition and expansion in the RTx430 genotype relative to the well-watered control and in comparison to the recovery of the BTx642 genotype.

By contrast, the leaf cell wall-related transcriptome did not have universal patterns across drought treatments. Aside from the pre-flowering drought induction treatment, which did not have many appreciable cell wall-related transcriptomic changes in either genotype, the genotypes had exaggerated differential expression for recovery and post-flowering drought (Supplementary Figure 3). The two genotypes diverged during the recovery period, with BTx642 having a greater number of glycosyltransferases (GTs) among the HVGs with increased expression relative to the watered control when compared to RTx430 lines (Supplementary Figure 4). Conversely, the RTx430 genotype experiencing post-flowering drought stress had a greater number of GTs with higher expression relative to the watered control when compared to BTx642 (Supplementary Figure 4). It is possible that this higher GT activity in older leaves, related to xylan, mixed-linkage glucan, and pectin biosynthesis, has a negative relationship with drought tolerance, as increased expression of these GTs occurred in the genotypes more susceptible to the drought stress or less efficient in recovery. Additionally, the RTx430 genotype experienced greater increase in expression of acetyl- and methyltransferases than BTx642 during post-flowering drought, but this dynamic was reversed during recovery from pre-flowering drought stress (Supplementary Figure 4). Thus, leaf cell wall-related transcriptomic changes appear to implicate different changes in polysaccharide abundance and structure. Sorghum from both genotypes that were re-watered after experiencing a pre-flowering drought stress had an increase in expression of putative wall expanding genes, such as expansins and XTHs (Supplementary Figure 5). Additionally, the same sorghum plants also had an increase in expression of putative secondary cell wall cellulose synthases (CESAs) and xylan backbone-synthesizing genes (Supplementary Figure 6). Taken together, the cell wall-related transcriptome of re-watered sorghum leaves after a pre-flowering drought induction suggests that these cell walls experience an increase in secondary cell wall deposition and expansion, with potentially modified polysaccharide structure in xylan and pectin. For sorghum experiencing a post-flowering drought stress, there was an observed increase in expression of putative secondary cell wall CESAs and acetyl- and methyltransferases (Supplementary Figures 4, 6). Interestingly, expression of expansins, XTHs, and pectin modifying enzymes (lyases and pectin methylesterases) appeared to decrease while expression of some GHs increased (Supplementary Figure 5). This suggests that post-flowering drought induction may result in increased deposition of the secondary cell wall in mature leaves with potentially affected pectin structure and esterification. Again, genes encoding putative pectin biosynthetic and modifying enzymes appear to be unusually highly represented, given that pectin is a very small portion of commelinid cell walls.

Based on the expression pattern of the majority of cell wall related HVGs over the course of the experiment (Supplementary Figures 7–16 and Supplementary Tables 1–10), we selected tissues from weeks 7 and fourteen for cell wall chemical analysis. During these weeks, plants have been subjected to four weeks of either pre-flowering drought stress, recovery, or post-flowering drought stress alongside the watered controls. Based on the differential expression analysis of cell wall-related HVGs, this should give the plant ample time to implement the downstream changes resulting from differential expression while also not occurring after the differential expression ceases.

### Cell Wall Composition Analysis

**FIGURE 2:**
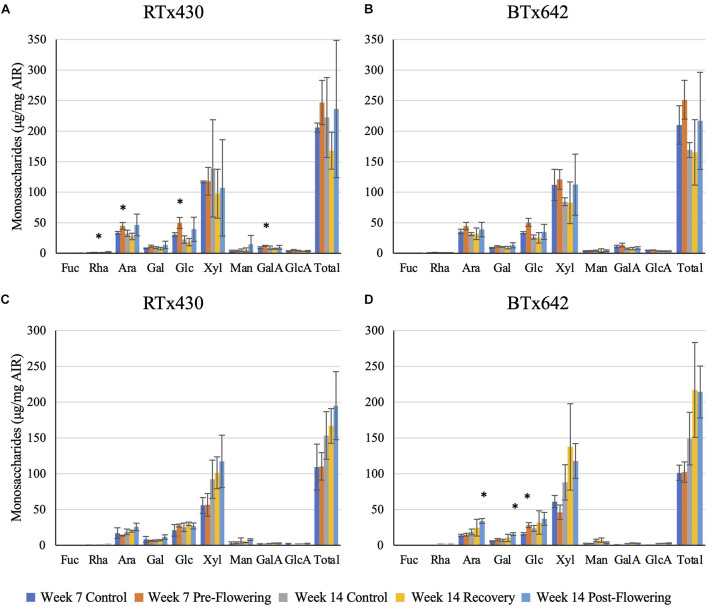
Matrix monosaccharide composition analysis. Monosaccharides released from a trifluoroacetic acid (TFA) hydrolysis of the alcohol insoluble residue (AIR) from leaves **(A,B)** and roots **(C,D)**. Data represents mean ± SD of three biological replicates per condition per genotype where biological replicates themselves consist of 10 pooled plants from the same planting block. Asterisks indicate *p* < 0.05 determined by Student’s *t*-test with the Benjamini–Hochberg correction.

Plants exposed to a pre-flowering drought stress had the greatest compositional change in cell wall monosaccharide composition relative to the well-watered condition in RTx430 (Figure 2). Young leaves had an increase in matrix monosaccharides, including rhamnose, arabinose, glucose, and galacturonic acid (62, 33, 62, and 37%, respectively, Figure 2A). BTx642 leaves experiencing pre-flowering drought stress also had an increase in matrix glucose, but the significance of the increase was lost when correcting for multiple comparisons (Figure 2B). Basal roots of BTx642 also showed an increase in matrix glucose (76%, Figure 2D) but demonstrated no additional monosaccharide compositional changes in the matrix sugars during pre-flowering drought stress.

**FIGURE 3:**
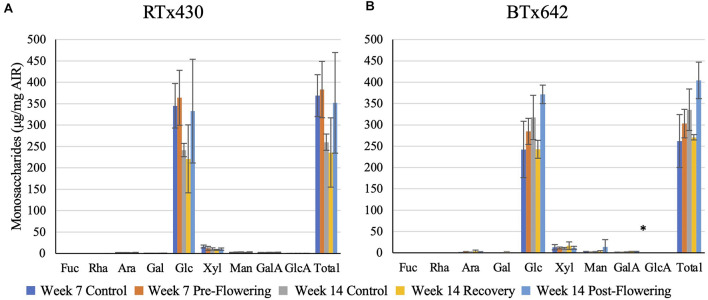
Cellulosic monosaccharide composition analysis. Monosaccharides released from a sulfuric acid hydrolysis of AIR already treated with TFA (matrix monosaccharides removed) from leaves **(A,B)**. Data represents mean ± SD of three biological replicates per condition per genotype where biological replicates themselves consist of ten pooled plants from the same planting block. Asterisk indicate *p* < 0.05 determined by Student’s *t*-test with the Benjamini–Hochberg correction.

Plants from either genotype recovering from a pre-flowering drought stress had no cell wall matrix or cellulosic compositional changes (Figures 2, 3). Plants exposed to a post-flowering drought stress had few cell wall compositional changes compared to plants exposed to pre-flowering drought stress (Figures 2, 3), with only BTx642 showing significant changes in the leaves and root. Leaves of BTx642 showed an increase in cellulose-associated galacturonic acid (38%, Figure 3B), while roots experienced an increase in matrix arabinose and galactose (81 and 130%, Figure 2D).

### Lignin

**FIGURE 4:**
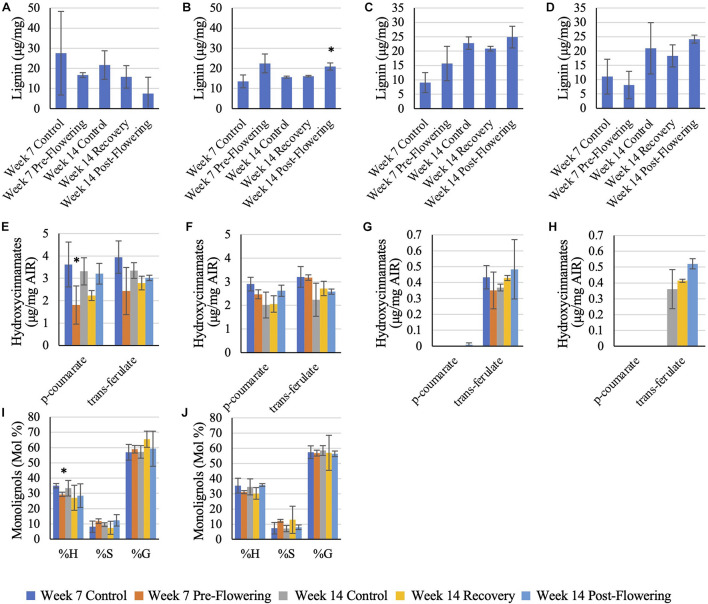
Lignin analysis of RTx430 **(A,C,E,G,I)** and BTx642 **(B,D,F,H,J)** leaves and roots. Released lignin from acetyl bromide treatment of leaves **(A,B)** and roots **(C,D)**. Data represents mean ± SD of three biological replicates per condition per genotype where biological replicates themselves consist of 10 pooled plants from the same planting block. Some replicates have been removed in this analysis, leaving two biological replicates rather than three, due to being extreme outliers. Monolignol relative composition of leaves **(E,F)** and roots **(G,H)**. Data represents mean ± SD of three biological replicates per condition per genotype where biological replicates themselves consist of ten pooled plants from the same planting block. Hydroxycinnamate content of leaf samples **(I,J)**, where data represents mean ± SD of three biological replicates per condition per genotype where biological replicates themselves consist of ten pooled plants from the same planting block. Several replicates had to be removed from the root samples (samples in which both *p*-coumarate and *trans*-ferulate were below the limit of detection). Standard deviation error bars, with data points without error bars representing points in which only one sample had detectable data. Asterisks indicate *p* < 0.05 from Student’s *t*-tests with the Benjamini–Hochman correction.

RTx430 plants showed no change in total lignin content across leaves and roots across the different irrigation conditions. However, RTx430 roots experiencing a pre-flowering drought stress showed a decrease in H units relative to total monolignol content, implying that the lignin in these roots may be more complex and resistant to degradation during a pre-flowering drought (Figure 4). RTx430 plants recovering from a pre-flowering drought showed a decrease in *p*-coumarate in the leaves and an increase in *trans*-ferulate in the roots, suggesting an alteration in cross-linking in the more extensible leaves and in the rigidifying roots.

BTx642 plants exposed to a post-flowering drought stress had an increase in total lignin in older leaves (Figure 4) but otherwise BTx642 plants showed no change in lignin or hydroxycinnamate content during drought conditions.

### Saccharification Efficiency

**FIGURE 5:**
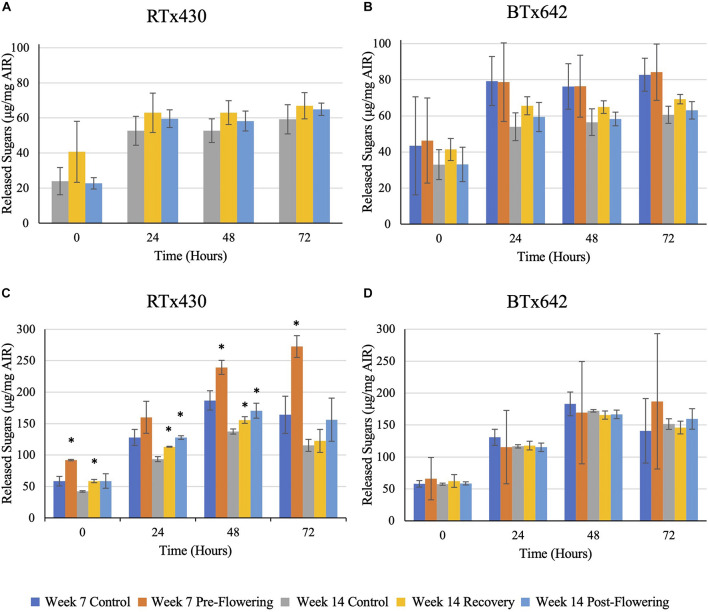
Saccharification efficiency with a hot-water pre-treatment of leaf samples. Samples were pre-treated using hot water before a 72-h digestion with CTec3. Reducing sugars were assayed via 3,5-dinitrosalicylic acid. Panels **(A,B)** refer to de-starched samples, **(C,D)** refer to AIR with starch intact. The data represents mean ± SD of three biological replicates per genotype per condition with samples themselves consisting of leaves pooled from ten plants within the same planting block. Standard deviation error bars, with asterisks indicate *p* < 0.05 using Student’s *t*-test with the Benjamini–Hochberg correction.

Drought stress had no negative implications on saccharification efficiency of leaves from either genotype, whether drought stress was induced pre- or post-flowering (Figure 5). Interestingly, when assaying the saccharification efficiency of leaves with starch content intact, an increase in the efficiency was observed under all drought conditions of RTx430, with up to 66, 39, and 36% increases for leaves experiencing pre-flowering drought, recovery, and post-flowering drought (Figure 5B). These increases in saccharification efficiency were lost when analyzing leaves with starch content enzymatically removed (Figure 5A).

## Discussion

### Transcriptome to Cell Wall

One of the biggest questions answered by this study is whether the transcriptome of droughted plants, when compared to well-watered plants, can accurately predict chemical changes within the plant cell wall. Concerning the cell wall-related HVGs, the transcriptome does not necessarily translate into measurable chemical wall changes. While some transcriptional changes resulted in expected cell wall changes, many were either correlated with no compositional changes or even the opposite of the expected changes. While many reported effects of drought stress on the plant cell wall are derived from transcriptomic studies that are rarely experimentally validated (Buchanan et al., 2005; Dugas et al., 2011; Le Gall et al., 2015; Tenhaken, 2015), this study demonstrates the need to validate the chemical and physical changes in the cell wall, especially as the tissues and conditions with the highest number of HVGs had the fewest changes in the wall, while the leaves experiencing pre-flowering drought induction had the fewest number of HVGs but the greatest compositional changes in the cell wall. These results support a growing body of literature reporting contrasting transcriptome results concerning drought stress, with *Brachypodium distachyon* demonstrating a wall-related transcriptome that did not always align with the metabolome in response to drought stress (Lenk et al., 2019).

Despite significant differences in expression of genes with cell wall-related GO terms, we detected few changes in the composition and saccharification efficiency of the cell walls of both *S. bicolor* cultivars in response to drought stress. This disconnect between transcriptome and wall composition can be explained by several different scenarios. The first scenario is that the HVGs encode enzymes that are minor contributors to wall biosynthesis and modification, with more active members of the gene families remaining constant under drought stress. Alternatively, there could be a large differential expression in these genes that is specific to a cell or tissue type, but the resulting change in composition is masked by that of other cell types. Importantly, such ‘minor contributor’ genes could still have an important role in drought response even if their role is not observable by bulk cell wall analysis of whole tissues or organs. Future studies, e.g., using reverse genetics with HVGs would help to clarify that. Another scenario could be that the enzymatic action is again occurring, but compositional changes are masked by the flux of carbon into and out of the cell wall. For example, it is possible that a great deal of new wall material is being deposited and/or modified, but either this new material or old material is being exuded from the wall for signaling and/or symbiotic processes with the rhizosphere (Xiao and Anderson, 2013; Anderson, 2016; Xu et al., 2018). Assuming that transcription does result in increased active enzyme levels, it is possible that the activity levels of the enzymes encoded by these HVGs are very low, or that the actual enzymatic activity is different from its putative function, as most of these encoded enzymes have not had any demonstrated activity in sorghum. A last scenario is one in which differential expression of cell wall-related genes, particularly those involved in biosynthesis and modification, could simply be the result of cells and their walls either ceasing to expand and grow, a known and visually obvious phenotype of progressive drought in leaves, or continuing to maintain extensibility in the roots despite reduced soil moisture availability. The ability to pinpoint the most likely set of scenarios will rely on further experiments exploring the composition and architecture of specific polysaccharides (xylan and pectin branching), exploring root sugar exudates, and exploring composition in a tissue and/or cell-specific context. Further useful experiments would also explore the cell wall-related proteome of these plants to understand whether differential expression also leads to differential enzyme accumulation.

Of the compositional changes that were observed, the most prominent changes happened to be in the matrix – namely, an increase in pectic rhamnose and galacturonic acid, arabinose, and glucose in the young leaves of RTx430 plants experiencing a pre-flowering drought stress and an increase in arabinose and galactose in the roots of BTx642 plants experiencing post-flowering drought stress. The changes in young leaves likely indicate an increase in pectin, arabinosylated xylan, and mixed-linkage glucan, although changes in abundance of arabinogalactan proteins could also be possible. Because these changes occurred in the pre-flowering drought-tolerant RTx430, but not BTx642, it is possible that these pectic and hemicellulosic differences contribute to pre-flowering drought tolerance. Root changes likely indicate an increase in arabinosylated xylan and either pectic galactan or arabinogalactan proteins. Despite not seeing an increase in any GALS1, PAGR, or GT31 homolog expression (Liwanag et al., 2012; Geshi et al., 2013; Stonebloom et al., 2016), we still observed an increase in galactose in the roots of BTx642. This could be due to an entirely different and unidentified galactosyltransferase, or alternatively it could be that known galactosyltransferase activity is regulated on the enzymatic level and thus is not observed in the transcriptome. In the same vein, we also did not observe any differential expression of GAUT or RRT1 homologs (Orfila et al., 2005; Atmodjo et al., 2011; Takenaka et al., 2018) during pre-flowering drought stress in RTx430 plants to account for changes in rhamnose and galacturonic acid. Pectin has been implicated in the plant drought response in the past (Micheli, 2001; An et al., 2008; Peaucelle et al., 2012; Liu et al., 2014), and pectic galactan and arabinan from rhamnogalacturonan I have been proposed to behave as “plasticizers” in drought and desiccation conditions for several different plants (Moore et al., 2008; Dinakar and Bartels, 2013). However, this focus on pectin’s role in the plant drought response has primarily been relegated to dicotyledons, in which pectins comprise a much greater percentage of the plant primary cell wall. These results coupled with that in the literature indicate that even in plant lineages where pectin is a very small portion of the plant primary and secondary cell wall, this polysaccharide may still play an outsize role in responding to and/or mitigating plant stress. Moreover, the over-representation of genes encoding pectin modifying enzymes amongst the HVGs in this study is striking, given that there are relatively few in sorghum, with only 16 pectic lyases and 35 carbohydrate esterases (including pectin methylesterases) (Rai et al., 2016). More work looking into pectic side chain composition, pectin esterification, and cleavage of pectic oligosaccharides in response to drought stress can help clarify the role of pectin in these processes.

An increase in matrix glucose was observed which often, but not always, correlated with a similar transcriptomic response in the CSLF gene family, which is known to encode mixed-linkage glucan synthases (Buckeridge et al., 2004). It is possible that this increase in glucose is in part derived from another cell wall polysaccharide, most likely amorphous cellulose that is released during hydrolysis with TFA. As with the pectin study, it is possible that either an unidentified glucosyltransferase is responsible for this increase in glucose or that the known glucosyltransferases are regulated tightly at the enzymatic level and the transcriptomic regulation does not reflect that. However, this is in contrast to previous literature in which mixed-linkage glucan decreased in the aerial biomass of other grasses, with a mixed response in the roots (Wakabayashi et al., 1997; Piro et al., 2003; Konno et al., 2008; Leucci et al., 2008; Rakszegi et al., 2013). MLG is deposited in the expanding cell wall and is correlated with increased wall extensibility (Buckeridge et al., 2004; Vega-Sanchez et al., 2012).

### Secondary Cell Wall Deposition

Transcriptomic changes in other putative cell wall biosynthetic genes such as CESAs, the GT61 family responsible for glycosylating the xylan backbone (Anders et al., 2012), and lignin biosynthetic enzymes (Schilmiller et al., 2009) were not reflected in cell wall compositional analyses. These upregulated genes could simply be a proxy for continued growth, including cell expansion and secondary cell wall deposition. Secondary cell wall CESAs were upregulated in older leaves of both cultivars in response to both post-flowering drought stress and pre-flowering drought stress recovery. This suggests an increase in secondary cell wall deposition in these older leaves as they either recover from drought stress or experience drought stress post-anthesis, likely from continued cell expansion and/or increased maturation. However, it does not suggest that the total secondary cell walls are more enriched in cellulose or lignin, which is borne out in the compositional wall analyses.

### Cell Wall Modification

Increased extensibility of root cell walls in response to low water potential linked to expansin abundance and activity has been explored and verified in the roots of maize seedlings exposed to drought or osmotic stress (Wu et al., 1996, 2001). Our own transcriptomic results align well with transcriptomic surveys of other grasses exposed to drought stress in relation to wall expanding genes such as expansins and XTHs (Buchanan et al., 2005; Dugas et al., 2011; Le Gall et al., 2015; Lenk et al., 2019). Expansin activity during drought stress results in more highly expansible cell walls, as has been shown in the apical domain of the maize root (Wu et al., 1996), and overexpression of a rose expansin in *Arabidopsis thaliana* has previously conferred drought tolerance (Lu et al., 2013). Interestingly, expansin expression decreased with distance from the root apex, implying that expansin activity during drought was focused to provide increased extensibility to the root apex, the portion of the root still expanding for deeper water reserves (Wu et al., 2001). Our data shows that expansin activity in the basal root is reduced during drought conditions, suggesting that in both genotypes, the basal root has decreased extensibility in response to drought stress. Interestingly, re-watered roots of the RTx430 pre-flowering drought tolerant genotype had increased expansin and XTH expression levels during the pre-flowering drought recovery period. As indicated by Xu et al. (2018), RTx430 appeared to have a faster recovery from pre-flowering drought stress, both in biomass recovery, but also in root microbiome recovery. This may be another indicator of pre-flowering drought tolerance, in which RTx430 is primed to resume cell division and expansion more rapidly after re-watering, which in turn gives a performance boost to the plant and its associated microbial communities via increased carbon flux.

### Biomass Conversion Efficiency

On a more practical level, this study indicates that drought stress does not impact saccharification efficiency of cell wall sugars in either cultivar and in fact can contribute to saccharification efficiency through starch accumulation in the RTx430 line (an increase of up to 66% in some assayed timepoints). As bioenergy crops like sorghum will likely be exposed to greater frequencies and durations of drought stress, this is welcome news, as saccharification penalties combined with potential biomass yield penalties would make bioenergy crops even less competitive in the energy market. From this study, combined with previously published data using this field trial (Xu et al., 2018; Varoquaux et al., 2019), we can conclude that the sorghum cultivars RTx430 and BTx642 experience no saccharification penalties under both pre-flowering and post-flowering drought stress. This study suggests that biorefineries do not need to be concerned with significant changes in biomass properties depending on drought patterns. One caveat to this conclusion is that the compositional and saccharification analyses were done on leaves, while stalks were not analyzed in this study. Since stalks are a major part of the aboveground biomass, it will be important to determine if the stalk cell walls and their saccharification are also largely unaffected in similar drought experiments.

### Summary and Significance

While there have been several studies detailing the transcriptomic differences of drought-stressed grasses (Buchanan et al., 2005; Dugas et al., 2011; Lenk et al., 2019), and several studies detailing cell wall changes in grasses affected by drought stress (Vincent et al., 2005; Emerson et al., 2014; Ottaiano et al., 2017; van der Weijde et al., 2017; El Hage et al., 2018; Hoover et al., 2018), this is the first study to detail the cell wall-related transcriptomic differences and the cell wall differences in a drought-tolerant C4 grass affected by several different drought stresses in the field. As with previous transcriptomic studies (Buchanan et al., 2005; Spollen et al., 2008; Dugas et al., 2011; Le Gall et al., 2015; Tenhaken, 2015; Rasheed et al., 2016; Lenk et al., 2019; Varoquaux et al., 2019), we see that GO terms and categories relating to the cell wall are significantly affected by drought stress, although the transcriptomic response to drought stress is large in sorghum, with more than 40% of expressed genes experiencing an effect on expression patterns relative to well-watered conditions (Varoquaux et al., 2019). In our own study, we noted a wide range of 15–117 cell wall related HVGs depending on the genotype and drought treatment, out of a total of 520 cell wall biosynthesis and modification genes already described in sorghum (Rai et al., 2016). Across these studies, genes thought to be involved in cell wall modification, particularly involving the extensibility of the wall, are particularly affected by drought. Conversely, recent studies on field-grown bioenergy grasses, including switchgrass, *Miscanthus*, and corn stover, demonstrate contrasting effects on cell wall composition and saccharification (Emerson et al., 2014; Ottaiano et al., 2017; Hoover et al., 2018). Despite the decrease in structural sugars found in these studies, sugar conversion was often unaffected or improved in plants exposed to drought stress, possibly due to a less recalcitrant cell wall. Our results, coupled with findings from these studies, indicate that drought stress does not result in biomass conversion penalties when using enzymatic hydrolysis, indicating that the cell wall is as or more accessible to enzymatic hydrolysis. This does not rule out the presence of microbial inhibitors. Our findings suggest that large compositional changes in the cell wall do not occur during drought stress, but this does not rule out structural changes in the cell wall and amongst its components. Importantly, drought, while predicted to have an increase in rigidification of tissues used for biomass processing, does not seem to affect the recalcitrance of the wall in the drought-tolerant *S. bicolor* RTx430 and BTx642 genotypes.

## Data Availability Statement

The original contributions presented in the study are included in the article/Supplementary Material, further inquiries can be directed to the corresponding author.

## Author Contributions

MM, JV, JD, RH, DC-D, PL, and EP designed the EpiCon field trial. NV and EP designed the transcriptomic experiments. NV generated and analyzed transcriptomic data. TS-D and HS designed the cell wall experiments. JO and MB generated lignin monomer composition data. TS-D generated and analyzed cell wall data. TS-D and HS wrote the manuscript. All authors approved the final manuscript.

## Conflict of Interest

The authors declare that the research was conducted in the absence of any commercial or financial relationships that could be construed as a potential conflict of interest.

## Publisher’s Note

All claims expressed in this article are solely those of the authors and do not necessarily represent those of their affiliated organizations, or those of the publisher, the editors and the reviewers. Any product that may be evaluated in this article, or claim that may be made by its manufacturer, is not guaranteed or endorsed by the publisher.

## Abbreviations

HVGs: highly variable genes
GO: gene ontology
GT: glycosyltransferase
GH: glycosyl hydrolase
XTH: xyloglucan endotransglucosylase/hydrolase
TFA: trifluoroacetic acid
CESAs: cellulose synthases
AIR: alcohol insoluble residue
CAZy: carbohydrate active enzymes
FDR: false discovery rate.
