# Supplementary material for: Cell Wall Compositions of Sorghum bicolor Leaves and Roots Remain Relatively Constant Under Drought Conditions
Source: Front Plant Sci. 2021 Nov 12;12:747225. doi: 10.3389/fpls.2021.747225 (PMC8632824; doi:10.3389/fpls.2021.747225)
Supplement: Supplementary file 1 [file Data_Sheet_1.zip › Supplementary Figures 1-6.pdf]

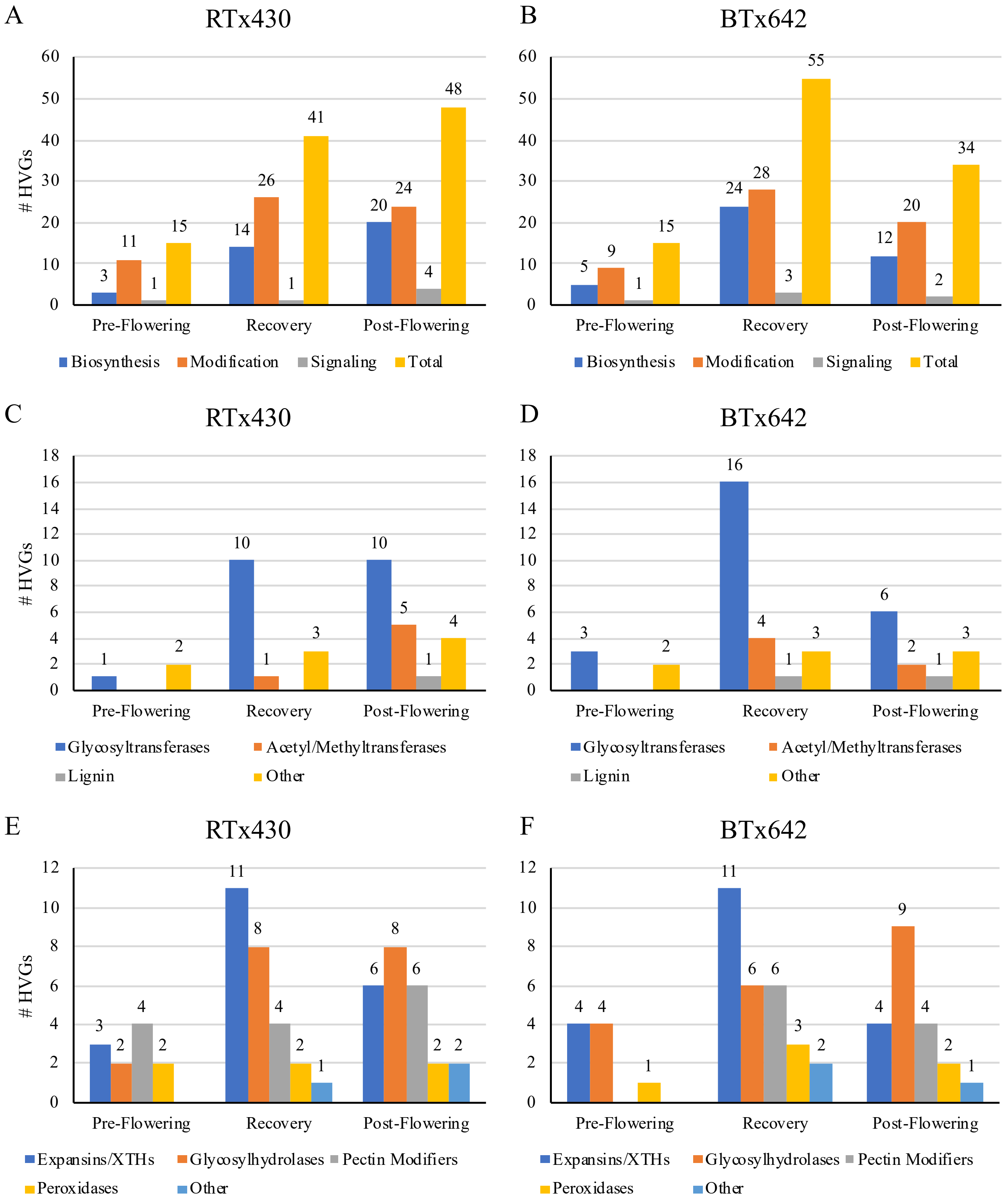

Figure S1. Cell wall-related transcriptome analysis of cell wall-related highly variable genes (HVGs) in leaves. Charts indicate the number of cell wall-related HVGs classified by putative function (A & B), with more resolution of the biosynthesis (C & D) and modification (E & F) categories.

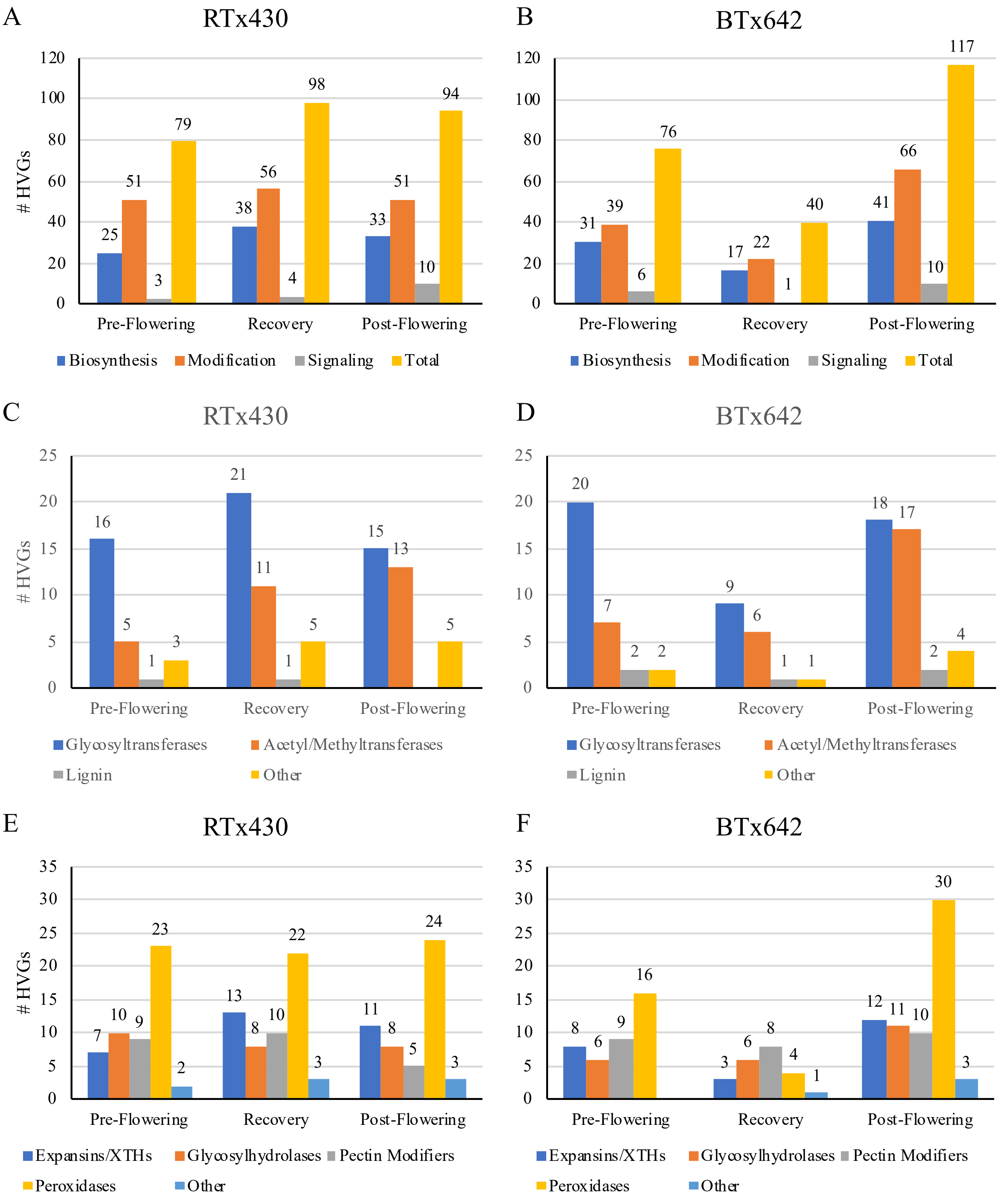

Figure S2. Cell wall-related transcriptome analysis of cell wall-related HVGs in roots. Charts indicate the number of cell wall-related HVGs classified by putative function (A & B), with more resolution of the biosynthesis (C & D) and modification (E & F) categories.

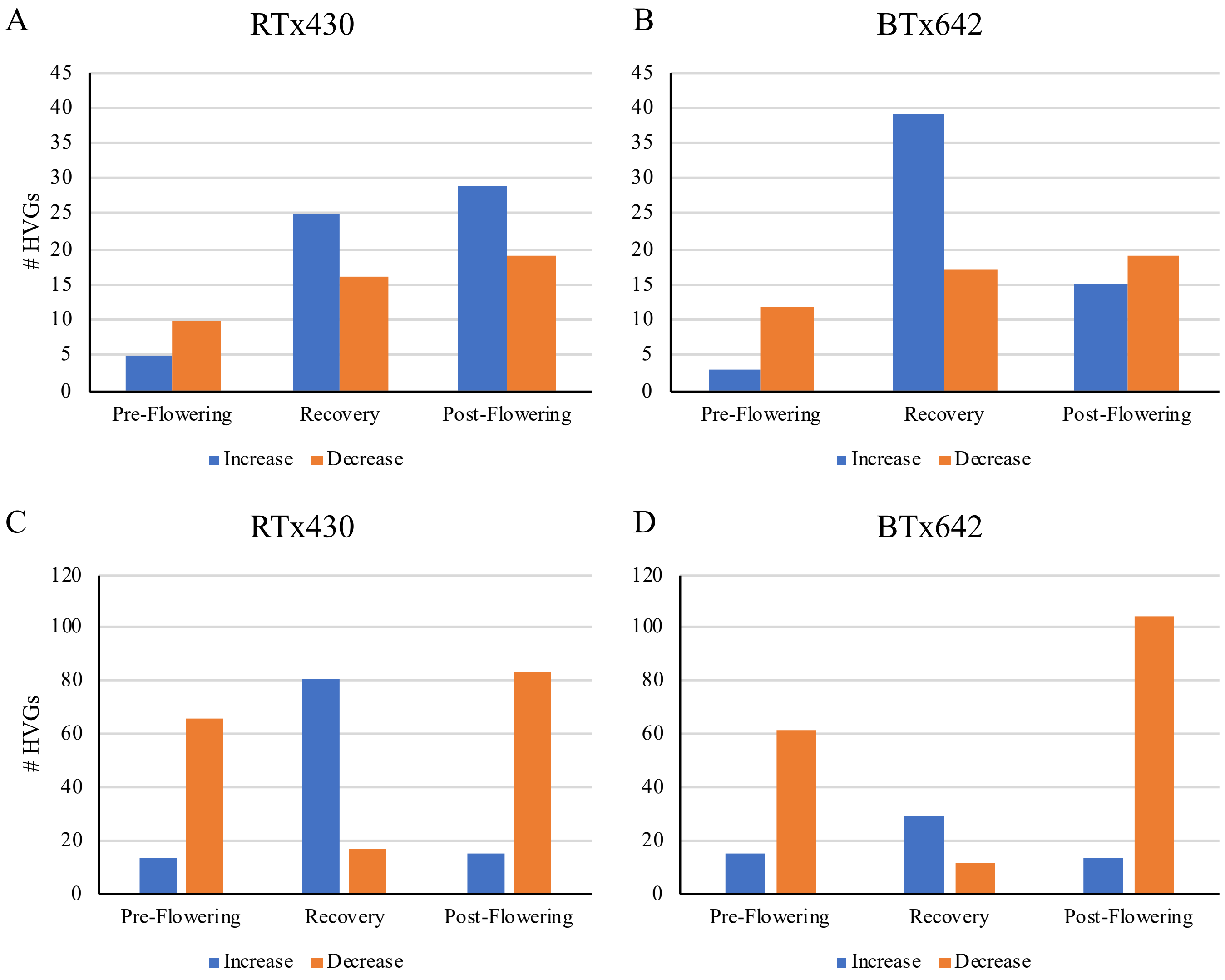

Figure S3. Number of cell wall-related HVGs with increased and decreased expression relative to the watered control in leaves (A, B) and roots (C, D).

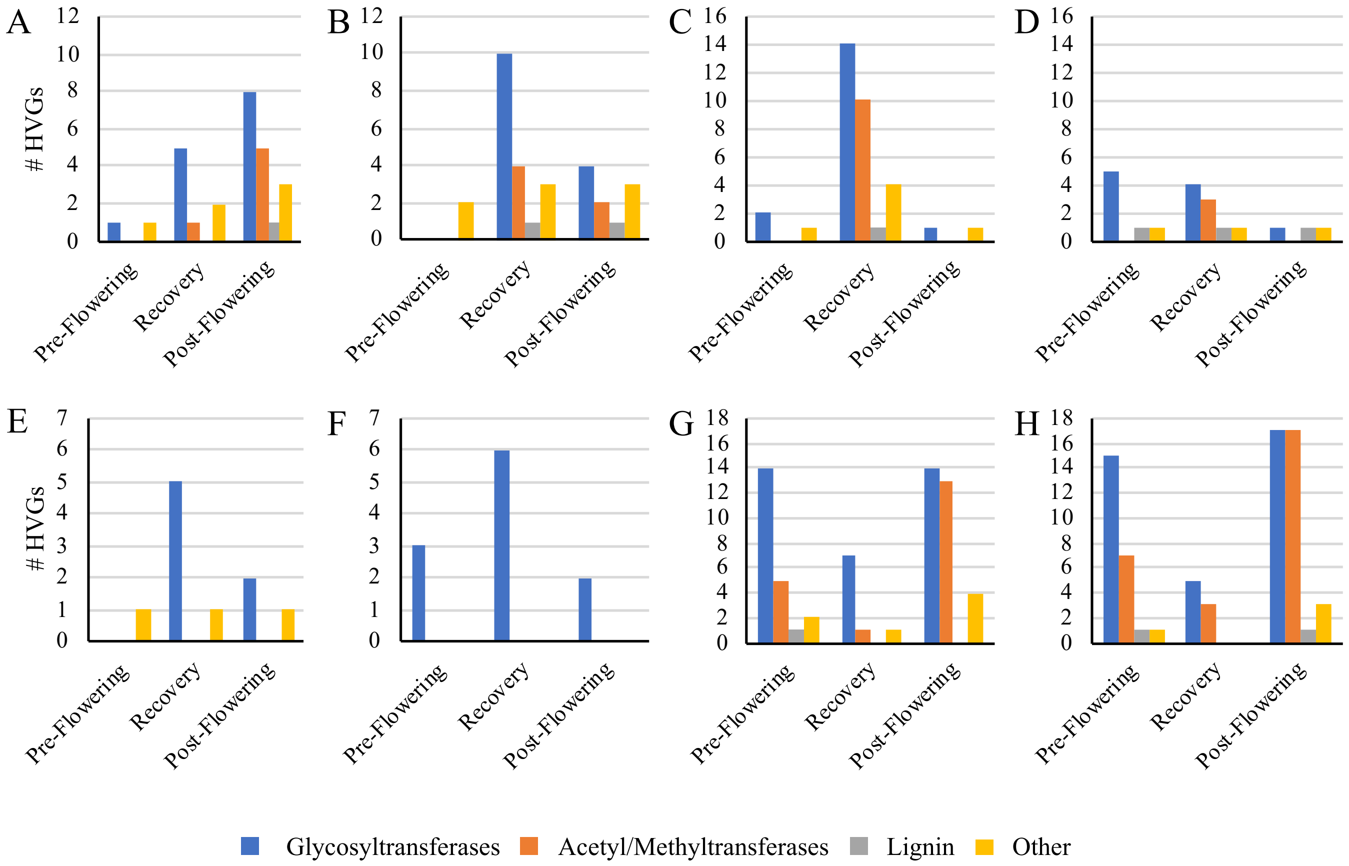

Figure S4. Biosynthesis category of HVGs in RTx430 (A, C, E, G) and BTx642 (B, D, F, H). Increased differential expression (A – D), decreased differential expression (E – H). Total number of HVGs pertaining to cell wall biosynthesis in leaves (A, B, E, F) and roots (C, D, G, H).

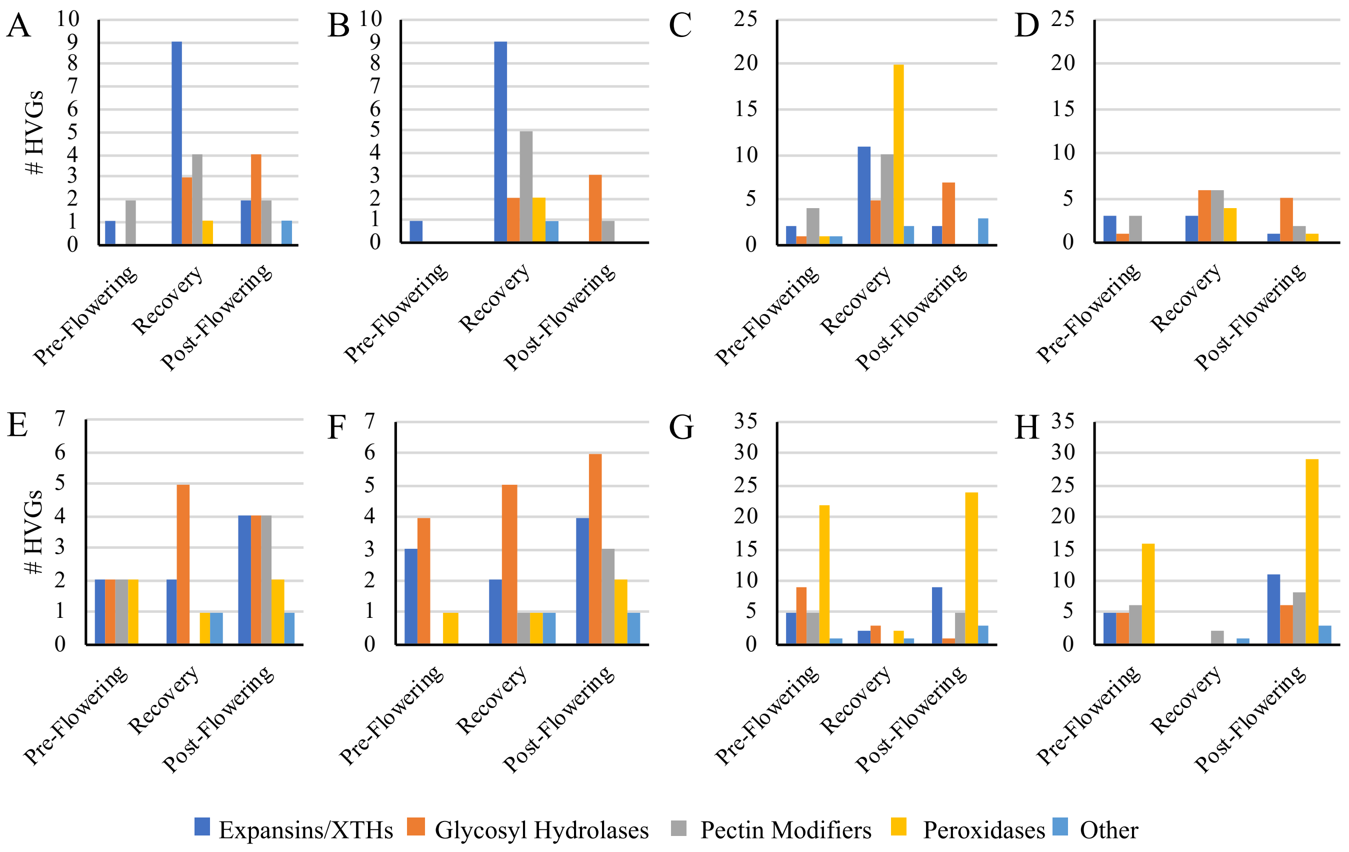

Figure S5. Modifications category of HVGs in leaves (A, B, E, F) and roots (C, D, G, H). RTx430 (A, C, E, G) and BTx642 (B, D, F, H) samples. Increased differential expression (A – D) and decreased differential expression (E – H).

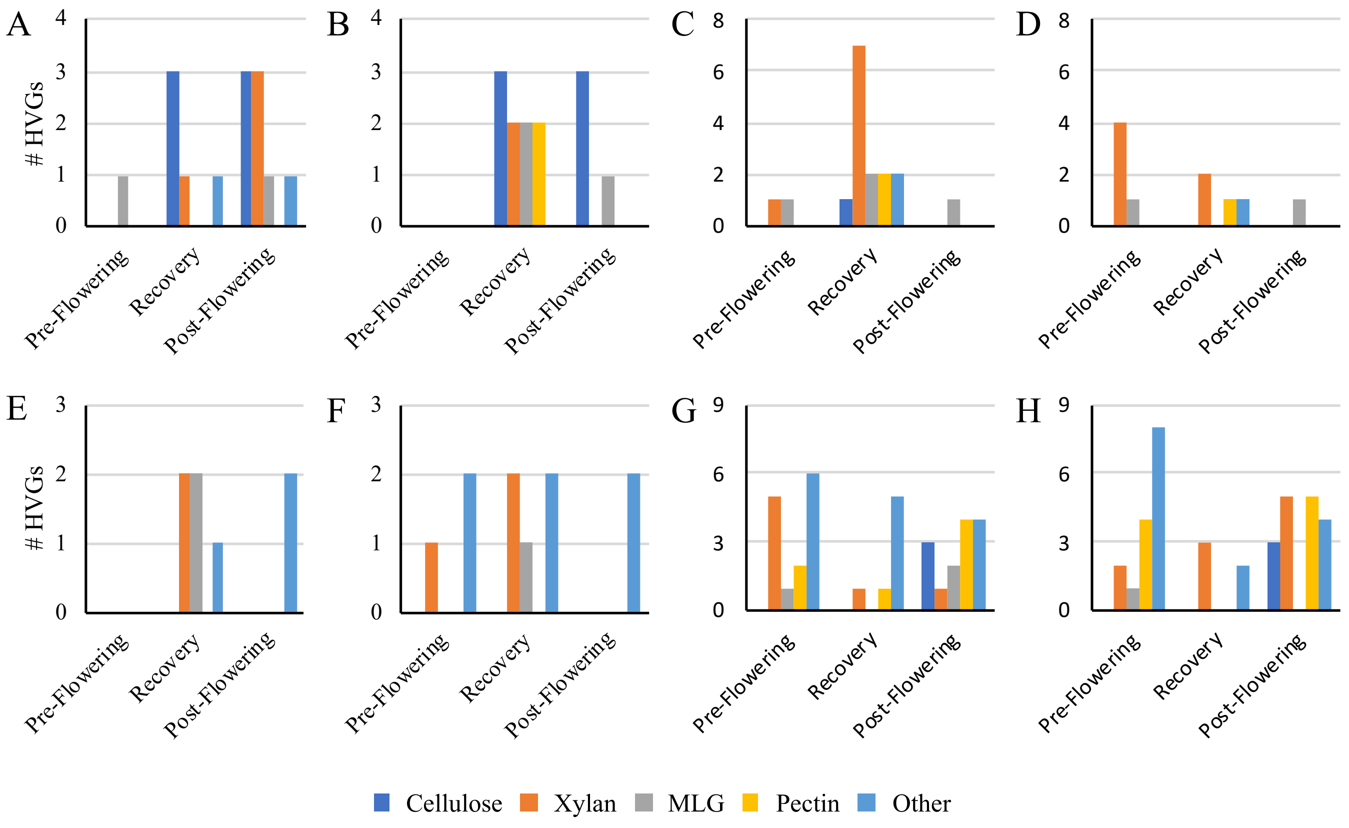

Figure S6. Glycosyltransferase category of HVGs in leaves (A, B, E, F) and root (C, D, G, H). RTx430 (A, C, E, G) and BTx642 (B, D, F, H) samples. Increased differential expression (A – D) and decreased differential expression (E – H).
