# Supplementary material for: Cell Wall Compositions of Sorghum bicolor Leaves and Roots Remain Relatively Constant Under Drought Conditions
Source: Front Plant Sci. 2021 Nov 12;12:747225. doi: 10.3389/fpls.2021.747225 (PMC8632824; doi:10.3389/fpls.2021.747225)
Supplement: Supplementary file 1 [file Data_Sheet_1.zip › Supplementary Figures 7-16 and Supplementary Tables 1-10.pdf]

Supplementary information: Cell wall compositions of *Sorghum bicolor* leaves and roots remain relatively constant under drought conditions

List of Figures

7 Cell-wall related genes (leaf) . . . 2
8 Cell-wall related genes (root) . . . 3
9 GO term 0009832 plant-type cell wall biogenesis (leaf) . . . 4
10 GO term 0009832 plant-type cell wall biogenesis (root) . . . 5
11 GO term 0042546 cell wall biogenesis (leaf) . . . 6
12 GO term 0042546 cell wall biogenesis (root) . . . 7
13 GO term 0071554 cell wall organization or biogenesis (leaf) . . . 8
14 GO term 0071554 cell wall organization or biogenesis (root) . . . 9
15 GO term 0071555 cell wall organization (leaf) . . . 10
16 GO term 0071555 cell wall organization (root) . . . 11

List of Tables

1 Highly variable cell-wall related genes (leaf) . . . 12
2 Highly variable cell-wall related genes (root) . . . 13
3 GO term “plant-type cell wall biogenesis” highly variable genes (leaf) . . . 14
4 GO term “plant-type cell wall biogenesis” highly variable genes (root) . . . 15
5 GO term “cell wall biogenesis” highly variable genes (leaf) . . . 16
6 GO term “cell wall biogenesis” highly variable genes (root) . . . 17
6 GO term “cell wall biogenesis” highly variable genes (root) . . . 18
7 GO term “cell wall organization or biogenesis” highly variable genes (leaf) . . . 19
7 GO term “cell wall organization or biogenesis” highly variable genes (leaf) . . . 20
7 GO term “cell wall organization or biogenesis” highly variable genes (leaf) . . . 21
8 GO term “cell wall organization or biogenesis” highly variable genes (root) . . . 22
8 GO term “cell wall organization or biogenesis” highly variable genes (root) . . . 23
8 GO term “cell wall organization or biogenesis” highly variable genes (root) . . . 24
8 GO term “cell wall organization or biogenesis” highly variable genes (root) . . . 25
8 GO term “cell wall organization or biogenesis” highly variable genes (root) . . . 26
9 GO term “cell wall organization” highly variable genes (leaf) . . . 27
9 GO term “cell wall organization” highly variable genes (leaf) . . . 28
10 GO term “cell wall organization” highly variable genes (root) . . . 29
10 GO term “cell wall organization” highly variable genes (root) . . . 30
10 GO term “cell wall organization” highly variable genes (root) . . . 31

1 Supplementary figures

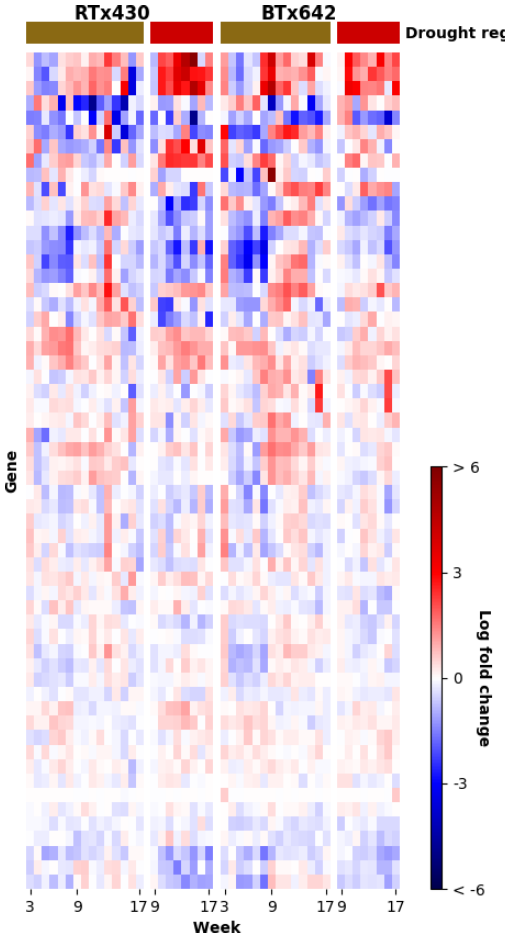

Figure S7: Log-fold change of cell-wall related gene (leaf). The average difference in the log expression of drought and control is shown via a heatmap, where the color scale corresponds to log-fold change values indicated in the accompanying legend, with blue corresponding to lower expression under drought, and red higher expression under drought. The columns indicate the weeks of sampling, divided into groups based on the two genotypes and then further divided into pre-flowering (brown) and post-flowering drought (red).

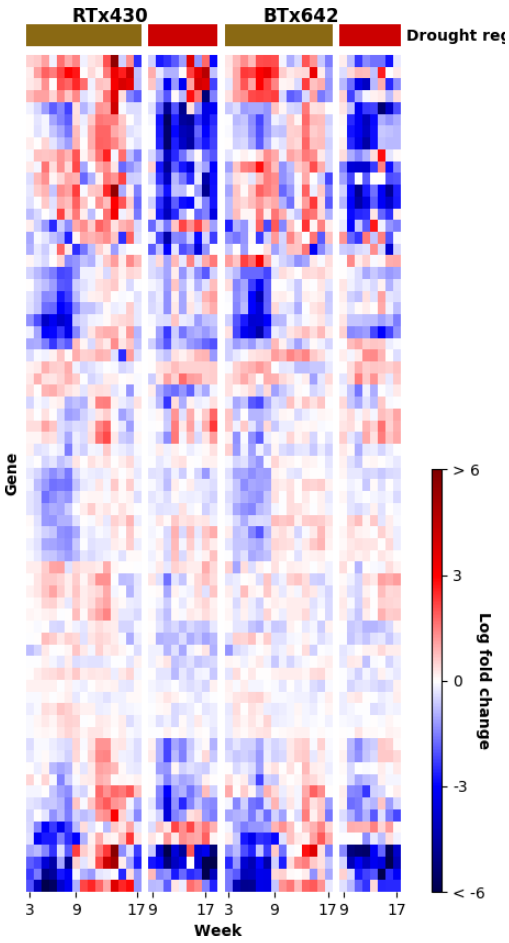

Figure S8: Log-fold change of cell-wall related gene (root). The average difference in the log expression of drought and control is shown via a heatmap, where the color scale corresponds to log-fold change values indicated in the accompanying legend, with blue corresponding to lower expression under drought, and red higher expression under drought. The columns indicate the weeks of sampling, divided into groups based on the two genotypes and then further divided into pre-flowering (brown) and post-flowering drought (red).

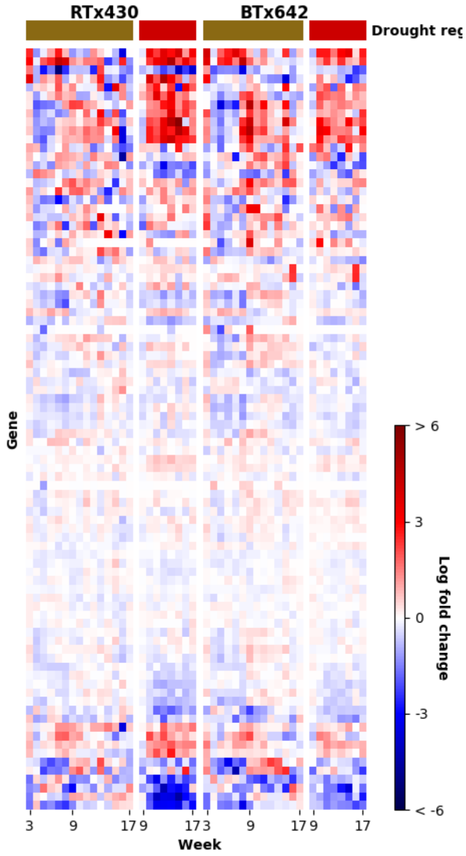

Figure S9: Log-fold change of cell-wall related gene (leaf). The average difference in the log expression of drought and control is shown via a heatmap, where the color scale corresponds to log-fold change values indicated in the accompanying legend, with blue corresponding to lower expression under drought, and red higher expression under drought. The columns indicate the weeks of sampling, divided into groups based on the two genotypes and then further divided into pre-flowering (brown) and post-flowering drought (red).

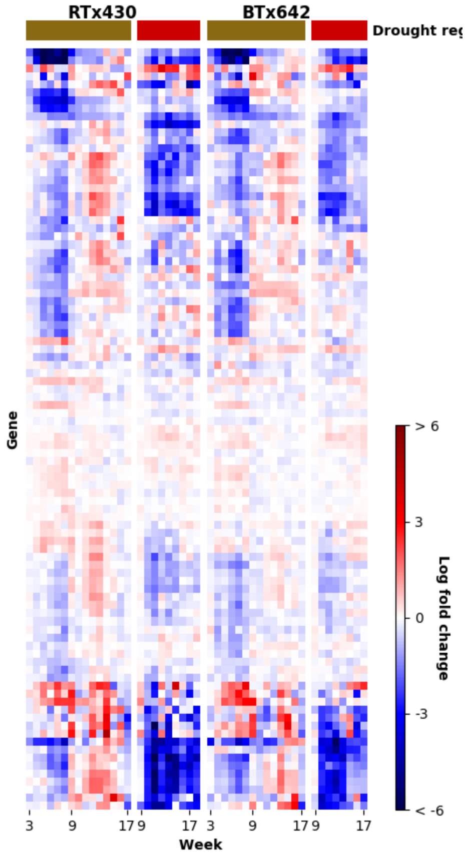

Figure S10: Log-fold change of cell-wall related gene (root). The average difference in the log expression of drought and control is shown via a heatmap, where the color scale corresponds to log-fold change values indicated in the accompanying legend, with blue corresponding to lower expression under drought, and red higher expression under drought. The columns indicate the weeks of sampling, divided into groups based on the two genotypes and then further divided into pre-flowering (brown) and post-flowering drought (red).

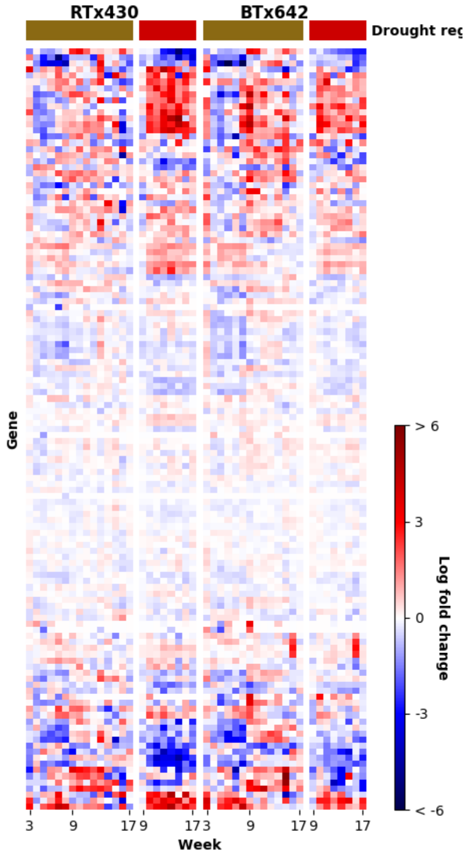

Figur**e S11: Log**-fold change of cell-wall related gene (leaf). The average difference in the log expression of drought and control is shown via a heatmap, where the color scale corresponds to log-fold change values indicated in the accompanying legend, with blue corresponding to lower expression under drought, and red higher expression under drought. The columns indicate the weeks of sampling, divided into groups based on the two genotypes and then further divided into pre-flowering (brown) and post-flowering drought (red).

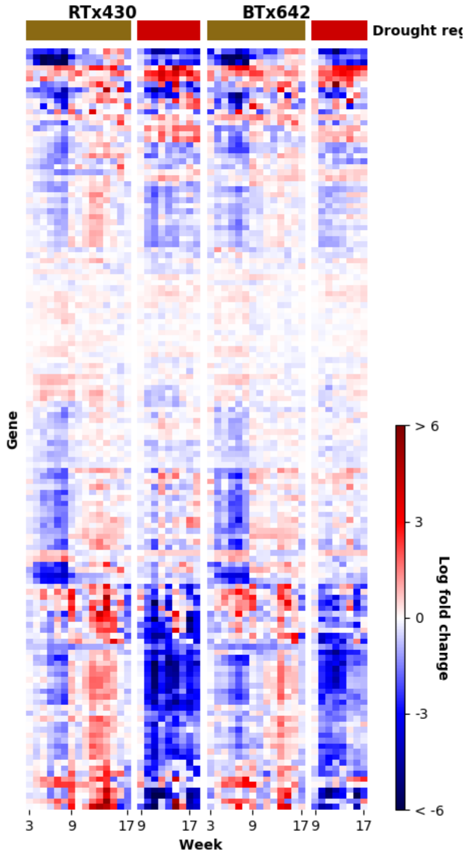

Figure S12: Log-fold change of cell-wall related gene (root). The average difference in the log expression of drought and control is shown via a heatmap, where the color scale corresponds to log-fold change values indicated in the accompanying legend, with blue corresponding to lower expression under drought, and red higher expression under drought. The columns indicate the weeks of sampling, divided into groups based on the two genotypes and then further divided into pre-flowering (brown) and post-flowering drought (red).

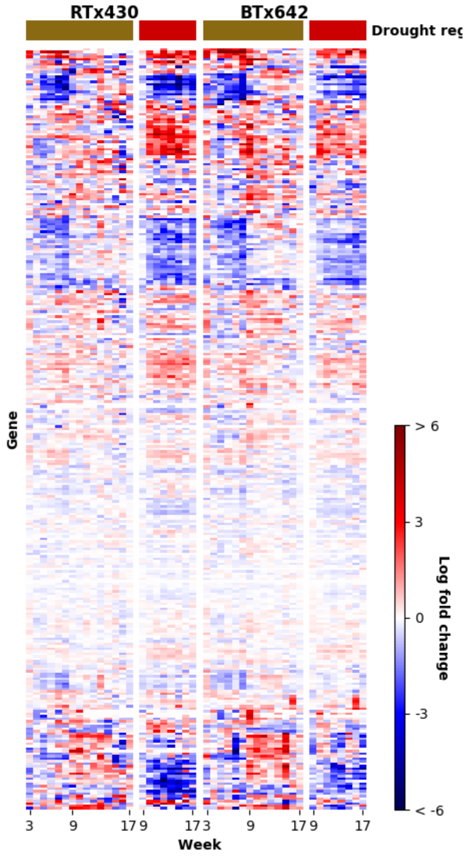

Figure S13: Log-fold change of cell-wall related gene (leaf). The average difference in the log expression of drought and control is shown via a heatmap, where the color scale corresponds to log-fold change values indicated in the accompanying legend, with blue corresponding to lower expression under drought, and red higher expression under drought. The columns indicate the weeks of sampling, divided into groups based on the two genotypes and then further divided into pre-flowering (brown) and post-flowering drought (red).

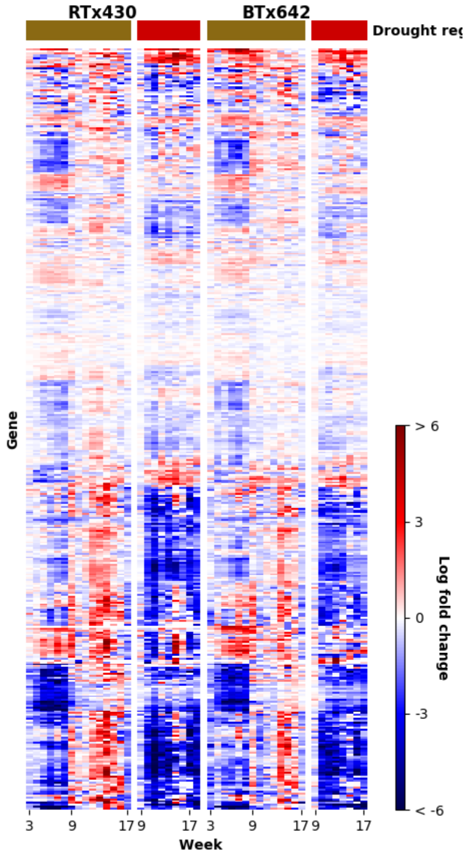

Figure S14: Log-fold change of cell-wall related gene (root). The average difference in the log expression of drought and control is shown via a heatmap, where the color scale corresponds to log-fold change values indicated in the accompanying legend, with blue corresponding to lower expression under drought, and red higher expression under drought. The columns indicate the weeks of sampling, divided into groups based on the two genotypes and then further divided into pre-flowering (brown) and post-flowering drought (red).

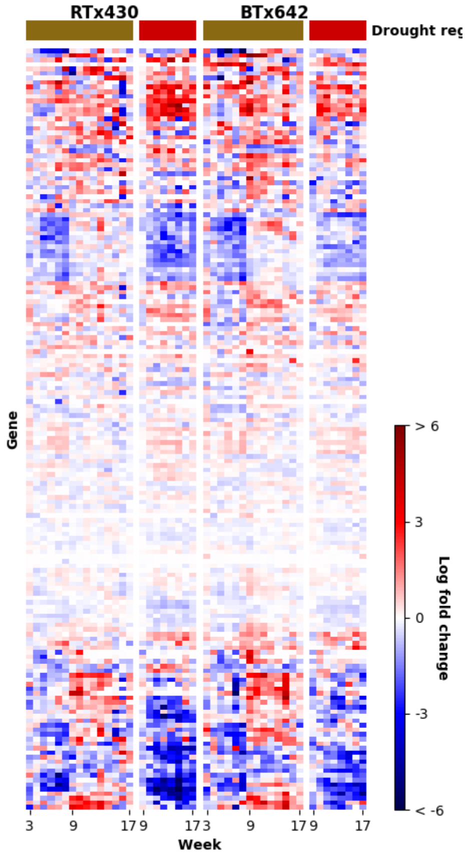

Figure S15: Log-fold change of cell-wall related gene (leaf). The average difference in the log expression of drought and control is shown via a heatmap, where the color scale corresponds to log-fold change values indicated in the accompanying legend, with blue corresponding to lower expression under drought, and red higher expression under drought. The columns indicate the weeks of sampling, divided into groups based on the two genotypes and then further divided into pre-flowering (brown) and post-flowering drought (red).

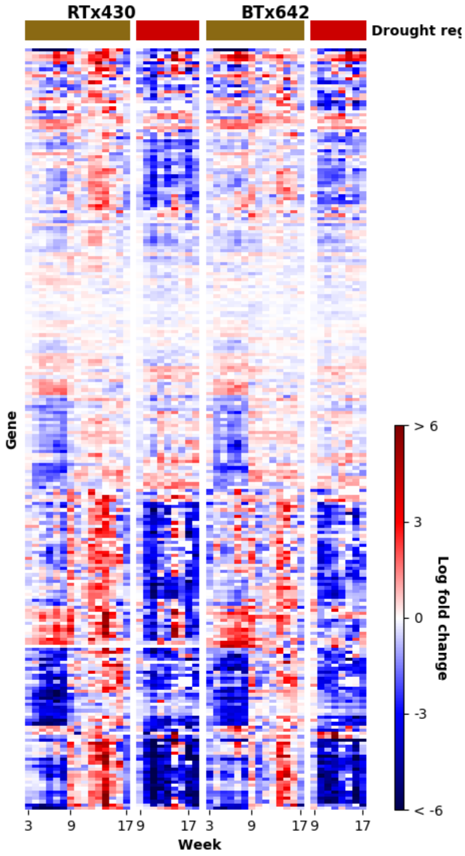

Figure S16: Log-fold change of cell-wall related gene (root). The average difference in the log expression of drought and control is shown via a heatmap, where the color scale corresponds to log-fold change values indicated in the accompanying legend, with blue corresponding to lower expression under drought, and red higher expression under drought. The columns indicate the weeks of sampling, divided into groups based on the two genotypes and then further divided into pre-flowering (brown) and post-flowering drought (red).

2 Supplementary tables

Table S1: Highly variable cell-wall related genes (leaf). List of genes found as highly variable for either genotypes in one of the following categories: Preflowering during the drought period, preflowering during the recovery period, or postflowering. The direction of the change is marked by a + (overexpressed in treatment) or - (underexpressed in treatment). The last two columns correspond to the closest homolog in arabidopsis and its description.

Gene	RTx430			BTx642			Ara. homolog	Desc.
	Pre.		Post.	Pre.		Post.		
	Drght	Rec.		Drght	Rec.			
Sobic.001G224300		+	+			+	CESA4, IRX5, NWS2	cellulose synthase A4
Sobic.002G205500		+	+		+	+	ATCESA7, CESA7, IRX3, MUR10	Cellulose synthase family protein
Sobic.003G296400			+		+	+	ATCESA8, CESA8, IRX1, LEW2	cellulose synthase family protein
Sobic.003G337400			+				ATC4H, C4H, CYP73A5, REF3	cinnamate-4-hydroxylase
Sobic.003G431100		-	+		-			Glycosyltransferase family 61 protein
Sobic.010G135300	+		+					Domain of unknown function (DUF23)
Sobic.002G094600					+		CESA6, E112, IXR2, PRC1	cellulose synthase 6
Sobic.002G118700		+			+		IXR2, E112, PRC1, CESA6	cellulose synthase 6
Sobic.002G171200					+		CSLD6, ATCSLD6, ATCSLD2, CSLD2	cellulose-synthase like D2,cellulose synthase-...
Sobic.003G094600					+			Glycosyltransferase family 61 protein
Sobic.007G050600					+		ATCSLD3, CSLD3, KJK	cellulose synthase-like D3
Sobic.010G230300					+			Galactosyltransferase family protein
Sobic.002G222800		-			-			Galactosyltransferase family protein
Sobic.004G320600		-			-			Glycosyltransferase family 61 protein
Sobic.010G008600				-			ATCSLD3, CSLD3, KJK	cellulose synthase-like D3
Sobic.010G030900				-				Glycosyltransferase family 61 protein

Table S2: Highly variable cell-wall related genes (root). List of genes found as highly variable for either genotypes in one of the following categories: Preflowering during the drought period, preflowering during the recovery period, or postflowering. The direction of the change is marked by a + (overexpressed in treatment) or - (underexpressed in treatment). The last two columns correspond to the closest homolog in arabidopsis and its description.

Sobic.001G224300			-			-	CESA4, IRX5, NWS2	cellulose synthase A4
Sobic.001G263300		+				-		Glycosyltransferase family 61 protein
Sobic.002G205500		+	-			-	ATCESA7, CESA7, IRX3, MUR10	Cellulose synthase family protein
Sobic.002G333900	-	-	-	-		-	ATCSLD3, CSLD3, KJK	cellulose synthase-like D3
Sobic.002G334000			-			-	ATCSLD5, CSLD5, SOS6	cellulose synthase-like D5
Sobic.002G334100		+	-	-		-	ATCSLD5, CSLD5, SOS6	cellulose synthase-like D5
Sobic.002G334200	-	+	-	-		-	ATCSLD5, CSLD5, SOS6	cellulose synthase-like D5
Sobic.003G095600	-	+		-		-		Glycosyltransferase family 61 protein
Sobic.003G095700						-		Glycosyltransferase family 61 protein
Sobic.003G296400			-			-	ATCESA8, CESA8, IRX1, LEW2	cellulose synthase family protein
Sobic.004G141200	-			-		-	ATC4H, C4H, CYP73A5, REF3	cinnamate-4-hydroxylase
Sobic.008G125700			-			-	ATCSLD5, CSLD5, SOS6	cellulose synthase-like D5
Sobic.010G152400		+	-			-		Glycosyltransferase family 61 protein
Sobic.010G152500		+				-		Glycosyltransferase family 61 protein
Sobic.003G095100			-		-			Glycosyltransferase family 61 protein
Sobic.010G196400		+	-					Domain of unknown function (DUF23)
Sobic.001G283400		+			+		ATCSLD3, CSLD3, KJK	cellulose synthase-like D3
Sobic.003G094700	+	+		+	+			Glycosyltransferase family 61 protein
Sobic.003G095500					+			Glycosyltransferase family 61 protein
Sobic.010G256400				+	-			Glycosyltransferase family 61 protein
Sobic.003G087700		+		+				Glycosyltransferase family 61 protein
Sobic.003G337400		+					ATC4H, C4H, CYP73A5, REF3	cinnamate-4-hydroxylase
Sobic.004G320600	-	+						Glycosyltransferase family 61 protein
Sobic.003G108500				+				Glycosyltransferase family 61 protein
Sobic.002G222800	-			-				Galactosyltransferase family protein
Sobic.003G363100				-				Glycosyltransferase family 29 (sialyl-transfera...
Sobic.010G008600				-			ATCSLD3, CSLD3, KJK	cellulose synthase-like D3
Sobic.010G030900	-			-				Glycosyltransferase family 61 protein
Sobic.003G095200	-							Glycosyltransferase family 61 protein
Sobic.003G431100	-							Glycosyltransferase family 61 protein

Table S3: GO term "plant-type cell wall biogenesis" highly variable genes (leaf). List of genes found as highly variable for either genotypes in one of the following categories: Preflowering during the drought period, preflowering during the recovery period, or postflowering. The direction of the change is marked by a + (overexpressed in treatment) or - (underexpressed in treatment). The last two columns correspond to the closest homolog in arabidopsis and its description.

Gene	RTx430			BTx642			Ara. homolog	Desc.
	Pre.		Post.	Pre.		Post.		
	Drght	Rec.		Drght	Rec.			
Sobic.001G224300		+	+			+	CESA4, IRX5, NWS2	cellulose synthase A4
Sobic.001G455700			+		+	+	ATMAP70-2, MAP70-2	microtubule-associated proteins 70-2
Sobic.002G205500		+	+		+	+	ATCESA7, CESA7, IRX3, MUR10	Cellulose synthase family protein
Sobic.002G368300			+	+		+	COBL4, IRX6	COBRA-like extracellular glycosyl-phosphatidyl...
Sobic.002G368600	+	+	+	+		+	ATRGP1, RGP1	reversibly glycosylated polypeptide 1
Sobic.003G251800			+			+	ANAC073, NAC073, SND2	NAC domain containing protein 73
Sobic.003G296400			+		+	+	ATCESA8, CESA8, IRX1, LEW2	cellulose synthase family protein
Sobic.004G124800		+	-			-	ATFT1, ATFUT1, FT1, MUR2	fucosyltransferase 1
Sobic.008G054100			-			-	ATFT1, ATFUT1, FT1, MUR2	fucosyltransferase 1
Sobic.001G038200			+				TBL33	TRICHOME BIREFRINGENCE-LIKE 33
Sobic.001G409100			+				IRX9	Nucleotide-diphospho-sugar transferases superf...
Sobic.006G242100			+				I14H, IRX14-L	Nucleotide-diphospho-sugar transferases superf...
Sobic.007G132600			+		-		AtMYB20, MYB20	myb domain protein 20
Sobic.002G427400	-	-	-				ATSEB1, COBL7, SEB1	COBRA-like protein-7 precursor
Sobic.007G166900		-	-		-		WAT1	Walls Are Thin 1
Sobic.001G399900					+		ATSEB1, COBL7, SEB1	COBRA-like protein-7 precursor
Sobic.003G250700					+		ATFLA11, FLA11	FASCICLIN-like arabinogalactan-protein 11
Sobic.007G018100					+		ANAC043, EMB2301, NST1	NAC (No Apical Meristem) domain transcriptiona...
Sobic.009G026101					+		IRX9	Nucleotide-diphospho-sugar transferases superf...
Sobic.003G410800		+					ATGUT1, GUT2, IRX10	Exostosin family protein
Sobic.009G241600		-						Zinc finger C-x8-C-x5-C-x3-H type family protein

Table S4: GO term "plant-type cell wall biogenesis" highly variable genes (root). List of genes found as highly variable for either genotypes in one of the following categories: Preflowering during the drought period, preflowering during the recovery period, or postflowering. The direction of the change is marked by a + (overexpressed in treatment) or - (underexpressed in treatment). The last two columns correspond to the closest homolog in arabidopsis and its description.

Gene	RTx430			BTx642			Ara. homolog	Desc.
	Pre.		Post.	Pre.		Post.		
	Drght	Rec.		Drght	Rec.			
Sobic.007G132600			+			+	AtMYB20, MYB20	myb domain protein 20
Sobic.001G086000						-	COB	COBRA-like extracellular glycosyl-phosphatidyl...
Sobic.001G224300			-			-	CESA4, IRX5, NWS2	cellulose synthase A4
Sobic.002G205500		+	-			-	ATCESA7, CESA7, IRX3, MUR10	Cellulose synthase family protein
Sobic.002G368600			-			-	ATRGP1, RGP1	reversibly glycosylated polypeptide 1
Sobic.003G236701			-	-		-		Zinc finger C-x8-C-x5-C-x3-H type family protein
Sobic.003G251800			-			-	ANAC073, NAC073, SND2	NAC domain containing protein 73
Sobic.003G296400			-			-	ATCESA8, CESA8, IRX1, LEW2	cellulose synthase family protein
Sobic.004G124800	-		-	-		-	ATFT1, ATFUT1, FT1, MUR2	fucosyltransferase 1
Sobic.006G160900		+	-			-	ANAC007, EMB2749, NAC007, VND4	NAC 007
Sobic.007G018100			-			-	ANAC043, EMB2301, NST1	NAC (No Apical Meristem) domain transcriptiona...
Sobic.007G039100			-			-	AtMYB103, MYB103	myb domain protein 103
Sobic.007G166900		-			-	-	WAT1	Walls Are Thin 1
Sobic.008G054100	-	-	-	-		-	ATFT1, ATFUT1, FT1, MUR2	fucosyltransferase 1
Sobic.008G112200			-			-	ATMYB46, MYB46	myb domain protein 46
Sobic.009G241600			-			-		Zinc finger C-x8-C-x5-C-x3-H type family protein
Sobic.010G082400						-	ATFT1, ATFUT1, FT1, MUR2	fucosyltransferase 1
Sobic.002G368300			-				COBL4, IRX6	COBRA-like extracellular glycosyl-phosphatidyl...
Sobic.005G034500			-					Leucine-rich repeat protein kinase family protein
Sobic.009G200200			-				ATTPS1, TPS1	trehalose-6-phosphate synthase
Sobic.007G177100	-			-	+		AtMYB32, MYB32	myb domain protein 32
Sobic.001G479800					-		GUX2, PGSIP3	plant glycogenin-like starch initiation protein 3
Sobic.004G125100	-	-		-	-		ATFT1, ATFUT1, FT1, MUR2	fucosyltransferase 1
Sobic.004G308600	-	-		-	-		ATFT1, ATFUT1, FT1, MUR2	fucosyltransferase 1
Sobic.001G336700		+					COBL4, IRX6	COBRA-like extracellular glycosyl-phosphatidyl...
Sobic.001G455700		+					ATMAP70-2, MAP70-2	microtubule-associated proteins 70-2
Sobic.004G231300		+					OSU1, QUA2, TSD2	S-adenosyl-L-methionine-dependent methyltransf...
Sobic.010G082000		+					ATFT1, ATFUT1, FT1, MUR2	fucosyltransferase 1
Sobic.010G155100		+					ANAC033, SMB	NAC (No Apical Meristem) domain transcriptiona...
Sobic.002G427400	-	-		-			ATSEB1, COBL7, SEB1	COBRA-like protein-7 precursor
Sobic.003G304600				+			ATRGP1, RGP1	reversibly glycosylated polypeptide 1
Sobic.003G250700				-			ATFLA11, FLA11	FASCICLIN-like arabinogalactan-protein 11

Table S5: GO term "cell wall biogenesis" highly variable genes (leaf). List of genes found as highly variable for either genotypes in one of the following categories: Preflowering during the drought period, preflowering during the recovery period, or postflowering. The direction of the change is marked by a + (overexpressed in treatment) or - (underexpressed in treatment). The last two columns correspond to the closest homolog in arabidopsis and its description.

Sobic.001G224300		+	+			+	CESA4, IRX5, NWS2	cellulose synthase A4
Sobic.001G406700			+			+	ESK1, TBL29	Plant protein of unknown function (DUF828)
Sobic.001G455700			+		+	+	ATMAP70-2, MAP70-2	microtubule-associated proteins 70-2
Sobic.002G205500		+	+		+	+	ATCESA7, CESA7, IRX3, MUR10	Cellulose synthase family protein
Sobic.002G368300			+	+		+	COBL4, IRX6	COBRA-like extracellular glycosyl-phosphatidyl...
Sobic.002G368600	+	+	+	+		+	ATRGP1, RGP1	reversibly glycosylated polypeptide 1
Sobic.003G251800			+			+	ANAC073, NAC073, SND2	NAC domain containing protein 73
Sobic.003G296400			+		+	+	ATCESA8, CESA8, IRX1, LEW2	cellulose synthase family protein
Sobic.004G124800		+	-			-	ATFT1, ATFUT1, FT1, MUR2	fucosyltransferase 1
Sobic.008G054100			-			-	ATFT1, ATFUT1, FT1, MUR2	fucosyltransferase 1
Sobic.010G246600		+	-		+	-	TCH4, XTH22	Xyloglucan endotransglucosylase/hydrolase fami...
Sobic.001G038200			+				TBL33	TRICHOME BIREFRINGENCE-LIKE 33
Sobic.001G409100			+				IRX9	Nucleotide-diphospho-sugar transferases superf...
Sobic.004G127200		-	+		-		ATXTH26, XTH26	xyloglucan endotransglucosylase/hydrolase 26
Sobic.006G242100			+				I14H, IRX14-L	Nucleotide-diphospho-sugar transferases superf...
Sobic.007G094900			+		+		TCH4, XTH22	Xyloglucan endotransglucosylase/hydrolase fami...
Sobic.007G132600			+		-		AtMYB20, MYB20	myb domain protein 20
Sobic.010G135300	+		+					Domain of unknown function (DUF23)
Sobic.002G427400	-	-	-				ATSEB1, COBL7, SEB1	COBRA-like protein-7 precursor
Sobic.007G166900		-	-		-		WAT1	Walls Are Thin 1
Sobic.001G309000		+			+		ATXTR8, XTH31, XTR8	xyloglucan endo-transglycosylase-related 8
Sobic.001G399900					+		ATSEB1, COBL7, SEB1	COBRA-like protein-7 precursor
Sobic.002G302000		+			+		XTH32	xyloglucan endotransglucosylase/hydrolase 32
Sobic.003G250700					+		ATFLA11, FLA11	FASCICLIN-like arabinogalactan-protein 11
Sobic.005G140001		+			+		EXGT-A4, XTH5	xyloglucan endotransglucosylase/hydrolase 5
Sobic.007G018100					+		ANAC043, EMB2301, NST1	NAC (No Apical Meristem) domain transcriptiona...
Sobic.009G026101					+		IRX9	Nucleotide-diphospho-sugar transferases superf...
Sobic.010G246500		+			+		TCH4, XTH22	Xyloglucan endotransglucosylase/hydrolase fami...
Sobic.010G246700		+			+		XTH25, XTR3	xyloglucan endotransglucosylase/hydrolase 25
Sobic.003G410800		+					ATGUT1, GUT2, IRX10	Exostosin family protein
Sobic.009G241600		-						Zinc finger C-x8-C-x5-C-x3-H type family protein
Sobic.002G324100				-			XTH8	xyloglucan endotransglucosylase/hydrolase 8
Sobic.006G205600	-			-			MERI-5, MERI5B, SEN4, XTH24	xyloglucan endotransglucosylase/hydrolase 24
Sobic.010G246400	-			-			TCH4, XTH22	Xyloglucan endotransglucosylase/hydrolase fami...

Table S6: GO term "cell wall biogenesis" highly variable genes (root). List of genes found as highly variable for either genotypes in one of the following categories: Preflowering during the drought period, preflowering during the recovery period, or postflowering. The direction of the change is marked by a + (overexpressed in treatment) or - (underexpressed in treatment). The last two columns correspond to the closest homolog in arabidopsis and its description.

Sobic.001G372500		+	+		+	+	ATUGE2, UGE2	UDP-D-glucose/UDP-D-galactose 4-epimerase 2
Sobic.007G094900	+	+	+	+	+	+	TCH4, XTH22	Xyloglucan endotransglucosylase/hydrolase fami...
Sobic.007G132600			+			+	AtMYB20, MYB20	myb domain protein 20
Sobic.001G038400			-	-		-	TBL34	TRICHOME BIREFRINGENCE-LIKE 34
Sobic.001G086000						-	COB	COBRA-like extracellular glycosyl-phosphatidyl...
Sobic.001G224300			-			-	CESA4, IRX5, NWS2	cellulose synthase A4
Sobic.001G406700			-			-	ESK1, TBL29	Plant protein of unknown function (DUF828)
Sobic.002G205500		+	-			-	ATCESA7, CESA7, IRX3, MUR10	Cellulose synthase family protein
Sobic.002G368600			-			-	ATRGP1, RGP1	reversibly glycosylated polypeptide 1
Sobic.003G236701			-	-		-		Zinc finger C-x8-C-x5-C-x3-H type family protein
Sobic.003G251800			-			-	ANAC073, NAC073, SND2	NAC domain containing protein 73
Sobic.003G296400			-			-	ATCESA8, CESA8, IRX1, LEW2	cellulose synthase family protein
Sobic.004G047600			-	-		-		Protein of unknown function (DUF579)
Sobic.004G124800	-		-	-		-	ATFT1, ATFUT1, FT1, MUR2	fucosyltransferase 1
Sobic.005G004900		+	-	-		-	TBL34	TRICHOME BIREFRINGENCE-LIKE 34
Sobic.005G140001		+	-			-	EXGT-A4, XTH5	xyloglucan endotransglucosylase/hydrolase 5
Sobic.006G160900		+	-			-	ANAC007, EMB2749, NAC007, VND4	NAC 007
Sobic.006G205500					+	-	XTH25, XTR3	xyloglucan endotransglucosylase/hydrolase 25
Sobic.006G205700		+	-			-	XTH16	xyloglucan endotransglucosylase/hydrolase 16
Sobic.007G018100			-			-	ANAC043, EMB2301, NST1	NAC (No Apical Meristem) domain transcriptiona...
Sobic.007G039100			-			-	AtMYB103, MYB103	myb domain protein 103
Sobic.007G086300			-			-	TCH4, XTH22	Xyloglucan endotransglucosylase/hydrolase fami...
Sobic.007G166900		-			-	-	WAT1	Walls Are Thin 1
Sobic.008G054100	-	-	-	-		-	ATFT1, ATFUT1, FT1, MUR2	fucosyltransferase 1
Sobic.008G112200			-			-	ATMYB46, MYB46	myb domain protein 46
Sobic.009G241600			-			-		Zinc finger C-x8-C-x5-C-x3-H type family protein
Sobic.010G082400						-	ATFT1, ATFUT1, FT1, MUR2	fucosyltransferase 1
Sobic.010G246600			+				TCH4, XTH22	Xyloglucan endotransglucosylase/hydrolase fami...
Sobic.002G302000		+	-				XTH32	xyloglucan endotransglucosylase/hydrolase 32
Sobic.002G368300			-				COBL4, IRX6	COBRA-like extracellular glycosyl-phosphatidyl...
Sobic.005G034500			-					Leucine-rich repeat protein kinase family protein
Sobic.009G200200			-				ATTPS1, TPS1	trehalose-6-phosphate synthase
Sobic.007G090436		+			+		TCH4, XTH22	Xyloglucan endotransglucosylase/hydrolase fami...
Sobic.007G177100	-			-	+		AtMYB32, MYB32	myb domain protein 32
Sobic.001G479800					-		GUX2, PGSIP3	plant glycogenin-like starch initiation protein 3
Sobic.004G125100	-	-		-	-		ATFT1, ATFUT1, FT1, MUR2	fucosyltransferase 1
Sobic.004G308600	-	-		-	-		ATFT1, ATFUT1, FT1, MUR2	fucosyltransferase 1
Sobic.001G336700		+					COBL4, IRX6	COBRA-like extracellular glycosyl-phosphatidyl...

Continued on next page

Table S6: GO term “cell wall biogenesis” highly variable genes (root). List of genes found as highly variable for either genotypes in one of the following categories: Preflowering during the drought period, preflowering during the recovery period, or postflowering. The direction of the change is marked by a + (overexpressed in treatment) or - (underexpressed in treatment). The last two columns correspond to the closest homolog in arabidopsis and its description.

Sobic.001G455700		+			ATMAP70-2, MAP70-2	microtubule-associated proteins 70-2
Sobic.004G126700		+			AtXTH12, XTH12	xyloglucan endotransglucosylase/hydrolase 12
Sobic.004G231300		+			OSU1, QUA2, TSD2	S-adenosyl-L-methionine-dependent methyltransf...
Sobic.007G090460		+			AtXTH13, XTH13	xyloglucan endotransglucosylase/hydrolase 13
Sobic.010G082000		+			ATFT1, ATFUT1, FT1, MUR2	fucosyltransferase 1
Sobic.010G155100		+			ANAC033, SMB	NAC (No Apical Meristem) domain transcriptiona...
Sobic.001G284600		-			ATXTH27, EXGT-A3, XTH27	endoxyloglucan transferase A3
Sobic.001G309000	-	-			ATXTR8, XTH31, XTR8	xyloglucan endo-transglycosylase-related 8
Sobic.002G427400	-	-	-		ATSEB1, COBL7, SEB1	COBRA-like protein-7 precursor
Sobic.003G304600			+		ATRGP1, RGP1	reversibly glycosylated polypeptide 1
Sobic.003G250700			-		ATFLA11, FLA11	FASCICLIN-like arabinogalactan-protein 11
Sobic.007G085600	-		-		XTH15, XTR7	xyloglucan endotransglucosylase/hydrolase 15

Table S7: GO term "cell wall organization or biogenesis" highly variable genes (leaf). List of genes found as highly variable for either genotypes in one of the following categories: Preflowering during the drought period, preflowering during the recovery period, or postflowering. The direction of the change is marked by a + (overexpressed in treatment) or - (underexpressed in treatment). The last two columns correspond to the closest homolog in arabidopsis and its description.

Sobic.001G027800		-	+		-	+		Glycosyl hydrolase superfamily protein
Sobic.001G224300		+	+			+	CESA4, IRX5, NWS2	cellulose synthase A4
Sobic.001G406700			+			+	ESK1, TBL29	Plant protein of unknown function (DUF828)
Sobic.001G455700			+		+	+	ATMAP70-2, MAP70-2	microtubule-associated proteins 70-2
Sobic.001G525000	+	+	+		+	+		Pectin lyase-like superfamily protein
Sobic.002G205500		+	+		+	+	ATCESA7, CESA7, IRX3, MUR10	Cellulose synthase family protein
Sobic.002G368300			+	+		+	COBL4, IRX6	COBRA-like extracellular glycosyl-phosphatidyl...
Sobic.002G368600	+	+	+	+		+	ATRGP1, RGP1	reversibly glycosylated polypeptide 1
Sobic.003G251800			+			+	ANAC073, NAC073, SND2	NAC domain containing protein 73
Sobic.003G296400			+		+	+	ATCESA8, CESA8, IRX1, LEW2	cellulose synthase family protein
Sobic.004G013800					+	+	TBL12	Plant protein of unknown function (DUF828)
Sobic.006G157700			+			+		Glycosyl hydrolase family protein
Sobic.006G235600		-			+	+	ATBXL4, XYL4	beta-D-xylosidase 4
Sobic.008G022500					+	+	GAUT7, LGT7	galacturonosyltransferase 7
Sobic.003G321200	-		-			-	ATPMEPCRA, PMEPCRA	methylesterase PCR A
Sobic.003G338801		+	-		+	-	AT-EXP1, AT-EXP1, ATEXPA1, ATHEXP ALPHA 1.2, EX...	expansin A1
Sobic.004G121900			-			-	ATEXP11, AT-EXPA11, ATHEXP ALPHA 1.14, EXP11, E...	expansin 11
Sobic.004G124800		+	-			-	ATFT1, ATFUT1, FT1, MUR2	fucosyltransferase 1
Sobic.004G208700			-			-	ATCHITIV, ATEP3, CHIV, EP3	homolog of carrot EP3-3 chitinase
Sobic.004G233700						-		Glycosyl hydrolase family protein
Sobic.006G116000						-	SVL1	SHV3-like 1
Sobic.006G132300			-			-	ATCHITIV, ATEP3, CHIV, EP3	homolog of carrot EP3-3 chitinase
Sobic.006G132400		-		-	-	-	ATCHITIV, ATEP3, CHIV, EP3	homolog of carrot EP3-3 chitinase
Sobic.006G132700		-			-	-	ATCHITIV, ATEP3, CHIV, EP3	homolog of carrot EP3-3 chitinase
Sobic.006G217900	-		-	-	+	-	FLS2	Leucine-rich receptor-like protein kinase fami...
Sobic.007G014200			-		-	-		Peroxidase superfamily protein
Sobic.008G054100			-			-	ATFT1, ATFUT1, FT1, MUR2	fucosyltransferase 1
Sobic.009G111000					-	-	ATPMEPCRB	Plant invertase/pectin methylesterase inhibito...
Sobic.009G173700		+	-			-	AT-EXP1, AT-EXP1, ATEXPA1, ATHEXP ALPHA 1.2, EX...	expansin A1
Sobic.010G005000						-		Pectin lyase-like superfamily protein
Sobic.010G128700	-		-	-		-		Peroxidase superfamily protein
Sobic.010G246600		+	-		+	-	TCH4, XTH22	Xyloglucan endotransglucosylase/hydrolase fami...
Sobic.001G038200			+				TBL33	TRICHOME BIREFRINGENCE-LIKE 33
Sobic.001G409100			+				IRX9	Nucleotide-diphospho-sugar transferases superf...
Sobic.003G085900			+				ATXYL1, TRG1, XYL1	alpha-xylosidase 1
Sobic.003G153200			+					Pectin lyase-like superfamily protein

Continued on next page

Table S7: GO term "cell wall organization or biogenesis" highly variable genes (leaf). List of genes found as highly variable for either genotypes in one of the following categories: Preflowering during the drought period, preflowering during the recovery period, or postflowering. The direction of the change is marked by a + (overexpressed in treatment) or - (underexpressed in treatment). The last two columns correspond to the closest homolog in arabidopsis and its description.

Sobic.003G240100		+	+				TBL21	TRICHOME BIREFRINGENCE-LIKE 21
Sobic.004G127200		-	+		-		ATXTH26, XTH26	xyloglucan endotransglucosylase/hydrolase 26
Sobic.004G197600			+				GPDL2, MRH5, SHV3	PLC-like phosphodiesterase family protein
Sobic.005G034100			+		+		TBL10	Plant protein of unknown function (DUF828)
Sobic.005G110460			+					Glycosyl hydrolase family protein
Sobic.006G242100			+				I14H, IRX14-L	Nucleotide-diphospho-sugar transferases superf...
Sobic.007G094900			+		+		TCH4, XTH22	Xyloglucan endotransglucosylase/hydrolase fami...
Sobic.007G132600			+		-		AtMYB20, MYB20	myb domain protein 20
Sobic.008G005100			+		+		TBL34	TRICHOME BIREFRINGENCE-LIKE 34
Sobic.010G135300	+		+					Domain of unknown function (DUF23)
Sobic.010G255500			+		-		EPC1	Nucleotide-diphospho-sugar transferases superf...
Sobic.002G427400	-	-	-				ATSEB1, COBL7, SEB1	COBRA-like protein-7 precursor
Sobic.003G223100			-		+			Pectin lyase-like superfamily protein
Sobic.004G113900			-					Pectin lyase-like superfamily protein
Sobic.007G166900		-	-		-		WAT1	Walls Are Thin 1
Sobic.010G273600	-		-	-			ATHCHIB, B-CHI, CHI-B, HCHIB, PR-3, PR3	basic chitinase
Sobic.K029900	-		-	-			ATHCHIB, B-CHI, CHI-B, HCHIB, PR-3, PR3	basic chitinase
Sobic.001G012300		+			+		CYP79B2	cytochrome P450, family 79, subfamily B, polyp...
Sobic.001G309000		+			+		ATXTR8, XTH31, XTR8	xyloglucan endo-transglycosylase-related 8
Sobic.001G399900					+		ATSEB1, COBL7, SEB1	COBRA-like protein-7 precursor
Sobic.002G094600					+		CESA6, E112, IXR2, PRC1	cellulose synthase 6
Sobic.002G302000		+			+		XTH32	xyloglucan endotransglucosylase/hydrolase 32
Sobic.003G250700					+		ATFLA11, FLA11	FASCICLIN-like arabinogalactan-protein 11
Sobic.003G282600					+		GAUT15	galacturonosyltransferase 15
Sobic.003G293800		+			+			Glycosyl hydrolase superfamily protein
Sobic.004G028700		+			+			Pectin lyase-like superfamily protein
Sobic.005G140001		+			+		EXGT-A4, XTH5	xyloglucan endotransglucosylase/hydrolase 5
Sobic.006G031900		+			+		ATEXP11, ATEXPA11, ATHEXP ALPHA 1.14, EXP11, E...	expansin 11
Sobic.007G018100					+		ANAC043, EMB2301, NST1	NAC (No Apical Meristem) domain transcriptiona...
Sobic.007G050600					+		ATCSLD3, CSLD3, KJK	cellulose synthase-like D3
Sobic.007G146200		+			+			Plant invertase/pectin methylesterase inhibito...
Sobic.009G026101					+		IRX9	Nucleotide-diphospho-sugar transferases superf...
Sobic.009G032600					+			Peroxidase superfamily protein
Sobic.009G243500		+			+			Pectin lyase-like superfamily protein
Sobic.010G078300					+		TBR	Plant protein of unknown function (DUF828)
Sobic.010G232500		+			+		RCI3, RCI3A	Peroxidase superfamily protein
Sobic.010G246500		+			+		TCH4, XTH22	Xyloglucan endotransglucosylase/hydrolase fami...
Sobic.010G246700		+			+		XTH25, XTR3	xyloglucan endotransglucosylase/hydrolase 25
Sobic.003G442500		-			-		ATCSLG3, CSLG3	cellulose synthase like G3
Sobic.004G237800		-			-		GAUT8, QUA1	Nucleotide-diphospho-sugar transferases superf...

Continued on next page

Table S7: GO term “cell wall organization or biogenesis” highly variable genes (leaf). List of genes found as highly variable for either genotypes in one of the following categories: Preflowering during the drought period, preflowering during the recovery period, or postflowering. The direction of the change is marked by a + (overexpressed in treatment) or - (underexpressed in treatment). The last two columns correspond to the closest homolog in arabidopsis and its description.

Sobic.006G132100					-		ATCHITIV, ATEP3, CHIV, EP3	homolog of carrot EP3-3 chitinase
Sobic.006G132500		-			-		ATCHITIV, ATEP3, CHIV, EP3	homolog of carrot EP3-3 chitinase
Sobic.001G516000		+					ATHCHIB, B-CHI, CHI-B, HCHIB, PR-3, PR3	basic chitinase
Sobic.003G410800		+					ATGUT1, GUT2, IRX10	Exostosin family protein
Sobic.006G132200		+		-			ATCHITIV, ATEP3, CHIV, EP3	homolog of carrot EP3-3 chitinase
Sobic.003G436800		-						Peroxidase superfamily protein
Sobic.009G216100		-						Pectin lyase-like superfamily protein
Sobic.009G241600		-						Zinc finger C-x8-C-x5-C-x3-H type family protein
Sobic.006G191700	+			+			ATEXP13, AT-EXPA13, ATHEXP ALPHA 1.22, EXP13, E...	expansin A13
Sobic.002G324100				-			XTH8	xyloglucan endotransglucosylase/hydrolase 8
Sobic.006G205600	-			-			MERI-5, MERI5B, SEN4, XTH24	xyloglucan endotransglucosylase/hydrolase 24
Sobic.010G008600				-			ATCSLD3, CSLD3, KJK	cellulose synthase-like D3
Sobic.010G246400	-			-			TCH4, XTH22	Xyloglucan endotransglucosylase/hydrolase fami...
Sobic.003G232600	+							Pectin lyase-like superfamily protein
Sobic.003G127100	-							Peroxidase superfamily protein

Table S8: GO term "cell wall organization or biogenesis" highly variable genes (root). List of genes found as highly variable for either genotypes in one of the following categories: Preflowering during the drought period, preflowering during the recovery period, or postflowering. The direction of the change is marked by a + (overexpressed in treatment) or - (underexpressed in treatment). The last two columns correspond to the closest homolog in arabidopsis and its description.

Sobic.001G012300	+	+		+	+	+	CYP79B2	cytochrome P450, family 79, subfamily B, polyp...
Sobic.001G027800		+	+		+	+		Glycosyl hydrolase superfamily protein
Sobic.001G372500		+	+		+	+	ATUGE2, UGE2	UDP-D-glucose/UDP-D-galactose 4-epimerase 2
Sobic.004G028700		+				+		Pectin lyase-like superfamily protein
Sobic.005G169500	+		+	+		+	GAUT6, GAUT4	galacturonosyltransferase 6,galacturonosyltran...
Sobic.006G132100			+			+	ATCHITIV, ATEP3, CHIV, EP3	homolog of carrot EP3-3 chitinase
Sobic.006G132300	+		+	+		+	ATCHITIV, ATEP3, CHIV, EP3	homolog of carrot EP3-3 chitinase
Sobic.007G094900	+	+	+	+	+	+	TCH4, XTH22	Xyloglucan endotransglucosylase/hydrolase fami...
Sobic.007G132600			+			+	AtMYB20, MYB20	myb domain protein 20
Sobic.009G032800	+	+			+	+	RCI3, RCI3A	Peroxidase superfamily protein
Sobic.009G216100		+			+	+		Pectin lyase-like superfamily protein
Sobic.010G273600		+	+			+	ATHCHIB, B-CHI, CHI-B, HCHIB, PR-3, PR3	basic chitinase
Sobic.K029900		+	+			+	ATHCHIB, B-CHI, CHI-B, HCHIB, PR-3, PR3	basic chitinase
Sobic.001G038300			-			-	TBL33	TRICHOME BIREFRINGENCE-LIKE 33
Sobic.001G038400			-	-		-	TBL34	TRICHOME BIREFRINGENCE-LIKE 34
Sobic.001G045800	-	+		-		-	ADPG2, PGAZAT	polygalacturonase abscission zone A. thaliana
Sobic.001G066900		+			+	-		Glycosyl hydrolase superfamily protein
Sobic.001G086000						-	COB	COBRA-like extracellular glycosyl-phosphatidyl...
Sobic.001G189000		+				-		Peroxidase superfamily protein
Sobic.001G189200						-		Peroxidase superfamily protein
Sobic.001G224300			-			-	CESA4, IRX5, NWS2	cellulose synthase A4
Sobic.001G237900			-	-		-	ATEXP11, AT-EXPA11, ATHEXP ALPHA 1.14, EXP11, E...	expansin 11
Sobic.001G238400						-	ATEXP11, AT-EXPA11, ATHEXP ALPHA 1.14, EXP11, E...	expansin 11
Sobic.001G242100	-	+	-	-		-	TBL19	TRICHOME BIREFRINGENCE-LIKE 19
Sobic.001G314000		+				-	RCI3, RCI3A	Peroxidase superfamily protein
Sobic.001G345300						-		Plant invertase/pectin methylesterase inhibito...
Sobic.001G406700			-			-	ESK1, TBL29	Plant protein of unknown function (DUF828)
Sobic.001G406901			-	-		-	TBL3	Plant protein of unknown function (DUF828)
Sobic.001G407000	-				-	-	PMR5, TBL44	Plant protein of unknown function (DUF828)
Sobic.001G499900		+				-	ATEXP11, AT-EXPA11, ATHEXP ALPHA 1.14, EXP11, E...	expansin 11
Sobic.002G003200		+	-			-		Peroxidase superfamily protein
Sobic.002G003700		+				-	RCI3, RCI3A	Peroxidase superfamily protein
Sobic.002G205500		+	-			-	ATCESA7, CESA7, IRX3, MUR10	Cellulose synthase family protein
Sobic.002G333900	-	-	-	-		-	ATCSLD3, CSLD3, KJK	cellulose synthase-like D3
Sobic.002G334000			-			-	ATCSLD5, CSLD5, SOS6	cellulose synthase-like D5

Continued on next page

Table S8: GO term “cell wall organization or biogenesis” highly variable genes (root). List of genes found as highly variable for either genotypes in one of the following categories: Preflowering during the drought period, preflowering during the recovery period, or postflowering. The direction of the change is marked by a + (overexpressed in treatment) or - (underexpressed in treatment). The last two columns correspond to the closest homolog in arabidopsis and its description.

Sobic.002G334100		+	-	-		-	ATCSLD5, CSLD5, SOS6	cellulose synthase-like D5
Sobic.002G334200	-	+	-	-		-	ATCSLD5, CSLD5, SOS6	cellulose synthase-like D5
Sobic.002G368600			-			-	ATRGP1, RGP1	reversibly glycosylated polypeptide 1
Sobic.002G391300		+	-			-		Peroxidase superfamily protein
Sobic.002G391400	-		-			-		Peroxidase superfamily protein
Sobic.002G391900	-	+	-			-		Peroxidase superfamily protein
Sobic.002G392000		+	-	-		-		Peroxidase superfamily protein
Sobic.002G392100	-	+	-	-		-		Peroxidase superfamily protein
Sobic.002G399400	-					-	TBL10	Plant protein of unknown function (DUF828)
Sobic.003G050300	-			-		-		Peroxidase superfamily protein
Sobic.003G140600	-	+	-	-		-		Peroxidase superfamily protein
Sobic.003G140700	-	+	-	-		-		Peroxidase superfamily protein
Sobic.003G142100						-	ATBXL2, BXL2	beta-xylosidase 2
Sobic.003G148300	-	+		-	+	-	ATPMEPCRB	Plant invertase/pectin methylesterase inhibito...
Sobic.003G152000	-		-			-		Peroxidase superfamily protein
Sobic.003G152200	-		-	-		-		Peroxidase superfamily protein
Sobic.003G167300	-		-			-		Peroxidase superfamily protein
Sobic.003G219300		+				-	TBR	Plant protein of unknown function (DUF828)
Sobic.003G232600		+				-		Pectin lyase-like superfamily protein
Sobic.003G236701			-	-		-		Zinc finger C-x8-C-x5-C-x3-H type family protein
Sobic.003G239700			-		-	-	TBL38	TRICHOME BIREFRINGENCE-LIKE 38
Sobic.003G251800			-			-	ANAC073, NAC073, SND2	NAC domain containing protein 73
Sobic.003G292100					-	-		Pectin lyase-like superfamily protein
Sobic.003G296400			-			-	ATCESA8, CESA8, IRX1, LEW2	cellulose synthase family protein
Sobic.003G338801			-			-	AT-EXP1, AT-EXP1, ATEXPA1, ATHEXP ALPHA 1.2, EX...	expansin A1
Sobic.004G013500		+	-			-	AtUGP2, UGP2	UDP-glucose pyrophosphorylase 2
Sobic.004G047600			-	-		-		Protein of unknown function (DUF579)
Sobic.004G113900						-		Pectin lyase-like superfamily protein
Sobic.004G124800	-		-	-		-	ATFT1, ATFUT1, FT1, MUR2	fucosyltransferase 1
Sobic.004G273900	-		-	-		-	RCI3, RCI3A	Peroxidase superfamily protein
Sobic.005G004900		+	-	-		-	TBL34	TRICHOME BIREFRINGENCE-LIKE 34
Sobic.005G005100	-	+	-	-		-	TBL34	TRICHOME BIREFRINGENCE-LIKE 34
Sobic.005G051500	-			-		-		Peroxidase superfamily protein
Sobic.005G140001		+	-			-	EXGT-A4, XTH5	xyloglucan endotransglucosylase/hydrolase 5
Sobic.005G211500	-	-		-		-	ATBXL2, BXL2	beta-xylosidase 2
Sobic.006G031900		+	-			-	ATEXP11, AT-EXPA11, ATHEXP ALPHA 1.14, EXP11, E...	expansin 11
Sobic.006G116000			-			-	SVL1	SHV3-like 1
Sobic.006G160900		+	-			-	ANAC007, EMB2749, NAC007, VND4	NAC 007
Sobic.006G205500					+	-	XTH25, XTR3	xyloglucan endotransglucosylase/hydrolase 25
Sobic.006G205700		+	-			-	XTH16	xyloglucan endotransglucosylase/hydrolase 16
Sobic.006G217900	-		-	-		-	FLS2	Leucine-rich receptor-like protein kinase fami...
Sobic.006G224500			-			-		Peroxidase superfamily protein
Sobic.006G235600			-	-		-	ATBXL4, XYL4	beta-D-xylosidase 4
Sobic.006G273300						-		Uncharacterised protein family (UPF0497)
Sobic.006G277500	-		-	-		-		Peroxidase superfamily protein

Continued on next page

Table S8: GO term "cell wall organization or biogenesis" highly variable genes (root). List of genes found as highly variable for either genotypes in one of the following categories: Preflowering during the drought period, preflowering during the recovery period, or postflowering. The direction of the change is marked by a + (overexpressed in treatment) or - (underexpressed in treatment). The last two columns correspond to the closest homolog in arabidopsis and its description.

Sobic.006G277550				-	+	-		Peroxidase superfamily protein
Sobic.006G277600						-		Peroxidase superfamily protein
Sobic.006G277700		+				-		Peroxidase superfamily protein
Sobic.006G277800	-		-			-		Peroxidase superfamily protein
Sobic.007G014200	-		-	-		-		Peroxidase superfamily protein
Sobic.007G018000	-			-		-	ATEXP25, AT-EXPA25, ATHEXP ALPHA 1.18, EXP25, E...	expansin A25
Sobic.007G018100			-			-	ANAC043, EMB2301, NST1	NAC (No Apical Meristem) domain transcriptiona...
Sobic.007G039100			-			-	AtMYB103, MYB103	myb domain protein 103
Sobic.007G086300			-			-	TCH4, XTH22	Xyloglucan endotransglucosylase/hydrolase fami...
Sobic.007G166900		-			-	-	WAT1	Walls Are Thin 1
Sobic.008G005100		+	-	-		-	TBL34	TRICHOME BIREFRINGENCE-LIKE 34
Sobic.008G005200		+		-		-	TBL34	TRICHOME BIREFRINGENCE-LIKE 34
Sobic.008G005800						-	TBL34	TRICHOME BIREFRINGENCE-LIKE 34
Sobic.008G054100	-	-	-	-		-	ATFT1, ATFUT1, FT1, MUR2	fucosyltransferase 1
Sobic.008G112200			-			-	ATMYB46, MYB46	myb domain protein 46
Sobic.008G125700			-			-	ATCSLD5, CSLD5, SOS6	cellulose synthase-like D5
Sobic.009G012100	-			-	-	-	ATHCHIB, B-CHI, CHI-B, HCHIB, PR-3, PR3	basic chitinase
Sobic.009G033500	-	-	-	-		-		Peroxidase superfamily protein
Sobic.009G035900	-	+				-		Chitinase family protein
Sobic.009G106500		+	-			-	TBL33	TRICHOME BIREFRINGENCE-LIKE 33
Sobic.009G186500		+	-			-	RCI3, RCI3A	Peroxidase superfamily protein
Sobic.009G203200						-		Pectin lyase-like superfamily protein
Sobic.009G241600			-			-		Zinc finger C-x8-C-x5-C-x3-H type family protein
Sobic.009G250800						-		Pectin lyase-like superfamily protein
Sobic.010G082400						-	ATFT1, ATFUT1, FT1, MUR2	fucosyltransferase 1
Sobic.010G096700		+				-	TBL27	TRICHOME BIREFRINGENCE-LIKE 27
Sobic.010G128700		+	-			-		Peroxidase superfamily protein
Sobic.010G155800			-			-	TBL38	TRICHOME BIREFRINGENCE-LIKE 38
Sobic.010G194500			-			-	ATEXP20, AT-EXPA20, ATHEXP ALPHA 1.23, EXP20, E...	expansin A20
Sobic.K026900	-	+	-	-		-		Peroxidase superfamily protein
Sobic.006G132400			+				ATCHITIV, ATEP3, CHIV, EP3	homolog of carrot EP3-3 chitinase
Sobic.006G132500	-		+		+		ATCHITIV, ATEP3, CHIV, EP3	homolog of carrot EP3-3 chitinase
Sobic.010G246600			+				TCH4, XTH22	Xyloglucan endotransglucosylase/hydrolase fami...
Sobic.001G238200	-		-	-			ATEXP11, AT-EXPA11, ATHEXP ALPHA 1.14, EXP11, E...	expansin 11
Sobic.002G302000		+	-				XTH32	xyloglucan endotransglucosylase/hydrolase 32
Sobic.002G368300			-				COBL4, IRX6	COBRA-like extracellular glycosyl-phosphatidyl...
Sobic.002G391200			-					Peroxidase superfamily protein
Sobic.002G416000			-				ATPME2, PME2	pectin methylesterase 2
Sobic.002G420100			-				GAUT7, LGT7	galacturonosyltransferase 7

Continued on next page

Table S8: GO term "cell wall organization or biogenesis" highly variable genes (root). List of genes found as highly variable for either genotypes in one of the following categories: Preflowering during the drought period, preflowering during the recovery period, or postflowering. The direction of the change is marked by a + (overexpressed in treatment) or - (underexpressed in treatment). The last two columns correspond to the closest homolog in arabidopsis and its description.

Sobic.003G148400		+	-				ATPME2, PME2	pectin methylesterase 2
Sobic.003G223100			-		-			Pectin lyase-like superfamily protein
Sobic.005G034500			-					Leucine-rich repeat protein kinase family protein
Sobic.005G229100		+	-		+		TBL27	TRICHOME BIREFRINGENCE-LIKE 27
Sobic.006G106900	-	+	-					Uncharacterised protein family (UPF0497)
Sobic.007G075600			-					Pectin lyase-like superfamily protein
Sobic.007G146200	-		-	-				Plant invertase/pectin methylesterase inhibito...
Sobic.008G114700			-				RCI3A, RCI3	Peroxidase superfamily protein
Sobic.009G055300		+	-		+		RCI3, RCI3A	Peroxidase superfamily protein
Sobic.009G200200			-				ATTPS1, TPS1	trehalose-6-phosphate synthase
Sobic.001G283400		+			+		ATCSLD3, CSLD3, KJK	cellulose synthase-like D3
Sobic.002G370300					+		QRT1	Pectin lyase-like superfamily protein
Sobic.003G050100	+				+			Pectin lyase-like superfamily protein
Sobic.003G141800					+			Pectin lyase-like superfamily protein
Sobic.003G153100	+	+		+	+			Pectin lyase-like superfamily protein
Sobic.003G437400					+			Peroxidase superfamily protein
Sobic.004G315000		+			+		TBR	Plant protein of unknown function (DUF828)
Sobic.005G110457					+			Glycosyl hydrolase family protein
Sobic.006G132700	-				+		ATCHITIV, ATEP3, CHIV, EP3	homolog of carrot EP3-3 chitinase
Sobic.007G090436		+			+		TCH4, XTH22	Xyloglucan endotransglucosylase/hydrolase fami...
Sobic.007G177100	-			-	+		AtMYB32, MYB32	myb domain protein 32
Sobic.009G111000	-			-	+		ATPMEPCRB	Plant invertase/pectin methylesterase inhibito...
Sobic.010G096400					+		TBL22	TRICHOME BIREFRINGENCE-LIKE 22
Sobic.K022500	-				+			Glycosyl hydrolase family protein
Sobic.001G479800					-		GUX2, PGSIP3	plant glycogenin-like starch initiation protein 3
Sobic.003G240400					-		TBL21	TRICHOME BIREFRINGENCE-LIKE 21
Sobic.004G125100	-	-		-	-		ATFT1, ATFUT1, FT1, MUR2	fucosyltransferase 1
Sobic.004G308600	-	-		-	-		ATFT1, ATFUT1, FT1, MUR2	fucosyltransferase 1
Sobic.001G085200		+					PDCB3	plasmodesmata callose-binding protein 3
Sobic.001G238000		+					ATEXP11, ATEXPA11, ATHEXP ALPHA 1.14, EXP11, E...	expansin 11
Sobic.001G336700		+					COBL4, IRX6	COBRA-like extracellular glycosyl-phosphatidyl...
Sobic.001G360400	-	+						Peroxidase superfamily protein
Sobic.001G455700		+					ATMAP70-2, MAP70-2	microtubule-associated proteins 70-2
Sobic.001G525000		+						Pectin lyase-like superfamily protein
Sobic.003G096300		+		+			HDG1, HD-GL2-1	homeodomain GLABROUS 1
Sobic.003G153200	+	+		+				Pectin lyase-like superfamily protein
Sobic.003G436800	-	+		-				Peroxidase superfamily protein
Sobic.004G126700		+					AtXTH12, XTH12	xyloglucan endotransglucosylase/hydrolase 12
Sobic.004G231300		+					OSU1, QUA2, TSD2	S-adenosyl-L-methionine-dependent methyltransf...
Sobic.004G313900	+	+					ATHDG11, EDT1, HDG11	homeodomain GLABROUS 11
Sobic.005G022500		+					ARAF, ARAF1, ASD1, ATASD1	alpha-L-arabinofuranosidase 1
Sobic.007G090460		+					AtXTH13, XTH13	xyloglucan endotransglucosylase/hydrolase 13
Sobic.009G033400		+					ATPA2, PA2	peroxidase 2
Sobic.009G243500		+						Pectin lyase-like superfamily protein
Sobic.010G082000		+					ATFT1, ATFUT1, FT1, MUR2	fucosyltransferase 1

Continued on next page

Table S8: GO term "cell wall organization or biogenesis" highly variable genes (root). List of genes found as highly variable for either genotypes in one of the following categories: Preflowering during the drought period, preflowering during the recovery period, or postflowering. The direction of the change is marked by a + (overexpressed in treatment) or - (underexpressed in treatment). The last two columns correspond to the closest homolog in arabidopsis and its description.

Sobic.010G155100		+					ANAC033, SMB	NAC (No Apical Meristem) domain transcriptiona...
Sobic.010G232500		+					RCI3, RCI3A	Peroxidase superfamily protein
Sobic.001G284600		-					ATXTH27, EXGT-A3, XTH27	endoxyloglucan transferase A3
Sobic.001G309000	-	-					ATXTR8, XTH31, XTR8	xyloglucan endo-transglycosylase-related 8
Sobic.001G516000	-	-					ATHCHIB, B-CHI, CHI-B, HCHIB, PR-3, PR3	basic chitinase
Sobic.002G237900	-	-		-			ATCSLE1, CSLE1	cellulose synthase like E1
Sobic.002G427400	-	-		-			ATSEB1, COBL7, SEB1	COBRA-like protein-7 precursor
Sobic.003G178000		-		-				Pectin lyase-like superfamily protein
Sobic.004G251800		-					TBL21	TRICHOME BIREFRINGENCE-LIKE 21
Sobic.006G186200		-					ATGT18, GT18	glycosyltransferase 18
Sobic.009G033300	-	-		-				Peroxidase superfamily protein
Sobic.001G499800	+			+			ATEXP11, AT-EXPA11, ATHEXP ALPHA 1.14, EXP11, E...	expansin 11
Sobic.003G304600				+			ATRGP1, RGP1	reversibly glycosylated polypeptide 1
Sobic.004G238801				+			AT-EXP1, AT-EXP1, ATEXPA1, ATHEXP ALPHA 1.2, EX...	expansin A1
Sobic.006G172000	+			+				Pectin lyase-like superfamily protein
Sobic.001G238300	-			-			ATEXP11, AT-EXPA11, ATHEXP ALPHA 1.14, EXP11, E...	expansin 11
Sobic.003G148700	-			-			TBL38	TRICHOME BIREFRINGENCE-LIKE 38
Sobic.003G250700				-			ATFLA11, FLA11	FASCICLIN-like arabinogalactan-protein 11
Sobic.003G321200	-			-			ATPMEPCRA, PMEPCRA	methylesterase PCR A
Sobic.004G208700	-			-			ATCHITIV, ATEP3, CHIV, EP3	homolog of carrot EP3-3 chitinase
Sobic.004G237800				-			GAUT8, QUA1	Nucleotide-diphospho-sugar transferases superf...
Sobic.007G085600	-			-			XTH15, XTR7	xyloglucan endotransglucosylase/hydrolase 15
Sobic.009G055100	-			-			RCI3, RCI3A	Peroxidase superfamily protein
Sobic.009G130100	-			-			ATHCHIB, B-CHI, CHI-B, HCHIB, PR-3, PR3	basic chitinase
Sobic.010G008600				-			ATCSLD3, CSLD3, KJK	cellulose synthase-like D3
Sobic.010G017600				-				Plant invertase/pectin methylesterase inhibito...
Sobic.003G320800	-						RCI3, RCI3A	Peroxidase superfamily protein
Sobic.003G442500	-						ATCSLG3, CSLG3	cellulose synthase like G3

Table S9: GO term "cell wall organization" highly variable genes (leaf). List of genes found as highly variable for either genotypes in one of the following categories: Preflowering during the drought period, preflowering during the recovery period, or postflowering. The direction of the change is marked by a + (overexpressed in treatment) or - (underexpressed in treatment). The last two columns correspond to the closest homolog in arabidopsis and its description.

Sobic.001G224300		+	+			+	CESA4, IRX5, NWS2	cellulose synthase A4
Sobic.001G525000	+	+	+		+	+		Pectin lyase-like superfamily protein
Sobic.002G205500		+	+		+	+	ATCESA7, CESA7, IRX3, MUR10	Cellulose synthase family protein
Sobic.002G368300			+	+		+	COBL4, IRX6	COBRA-like extracellular glycosyl-phosphatidyl...
Sobic.003G296400			+		+	+	ATCESA8, CESA8, IRX1, LEW2	cellulose synthase family protein
Sobic.008G022500					+	+	GAUT7, LGT7	galacturonosyltransferase 7
Sobic.003G321200	-		-			-	ATPMEPCRA, PMEPCRA	methylesterase PCR A
Sobic.003G338801		+	-		+	-	AT-EXP1, AT-EXP1, ATEXPA1, ATHEXP ALPHA 1.2, EX...	expansin A1
Sobic.004G121900			-			-	ATEXP11, AT-EXPA11, ATHEXP ALPHA 1.14, EXP11, E...	expansin 11
Sobic.006G116000						-	SVL1	SHV3-like 1
Sobic.006G217900	-		-	-	+	-	FLS2	Leucine-rich receptor-like protein kinase fami...
Sobic.007G014200			-		-	-		Peroxidase superfamily protein
Sobic.009G111000					-	-	ATPMEPCRB	Plant invertase/pectin methylesterase inhibito...
Sobic.009G173700		+	-			-	AT-EXP1, AT-EXP1, ATEXPA1, ATHEXP ALPHA 1.2, EX...	expansin A1
Sobic.010G005000						-		Pectin lyase-like superfamily protein
Sobic.010G128700	-		-	-		-		Peroxidase superfamily protein
Sobic.010G246600		+	-		+	-	TCH4, XTH22	Xyloglucan endotransglucosylase/hydrolase fami...
Sobic.003G153200			+					Pectin lyase-like superfamily protein
Sobic.004G127200		-	+		-		ATXTH26, XTH26	xyloglucan endotransglucosylase/hydrolase 26
Sobic.004G197600			+				GPDL2, MRH5, SHV3	PLC-like phosphodiesterase family protein
Sobic.007G094900			+		+		TCH4, XTH22	Xyloglucan endotransglucosylase/hydrolase fami...
Sobic.010G255500			+		-		EPC1	Nucleotide-diphospho-sugar transferases superf...
Sobic.002G427400	-	-	-				ATSEB1, COBL7, SEB1	COBRA-like protein-7 precursor
Sobic.003G223100			-		+			Pectin lyase-like superfamily protein
Sobic.004G113900			-					Pectin lyase-like superfamily protein
Sobic.001G012300		+			+		CYP79B2	cytochrome P450, family 79, subfamily B, polyp...
Sobic.001G309000		+			+		ATXTR8, XTH31, XTR8	xyloglucan endo-transglycosylase-related 8
Sobic.001G399900					+		ATSEB1, COBL7, SEB1	COBRA-like protein-7 precursor
Sobic.002G094600					+		CESA6, E112, IXR2, PRC1	cellulose synthase 6
Sobic.002G302000		+			+		XTH32	xyloglucan endotransglucosylase/hydrolase 32
Sobic.003G282600					+		GAUT15	galacturonosyltransferase 15
Sobic.004G028700		+			+			Pectin lyase-like superfamily protein
Sobic.005G140001		+			+		EXGT-A4, XTH5	xyloglucan endotransglucosylase/hydrolase 5
Sobic.006G031900		+			+		ATEXP11, AT-EXPA11, ATHEXP ALPHA 1.14, EXP11, E...	expansin 11
Sobic.007G050600					+		ATCSLD3, CSLD3, KJK	cellulose synthase-like D3
Sobic.007G146200		+			+			Plant invertase/pectin methylesterase inhibito...
Sobic.009G032600					+			Peroxidase superfamily protein

Continued on next page

Table S9: GO term "cell wall organization" highly variable genes (leaf). List of genes found as highly variable for either genotypes in one of the following categories: Preflowering during the drought period, preflowering during the recovery period, or postflowering. The direction of the change is marked by a + (overexpressed in treatment) or - (underexpressed in treatment). The last two columns correspond to the closest homolog in arabidopsis and its description.

Sobic.009G243500		+		+		Pectin lyase-like superfamily protein
Sobic.010G232500		+		+	RCI3, RCI3A	Peroxidase superfamily protein
Sobic.010G246500		+		+	TCH4, XTH22	Xyloglucan endotransglucosylase/hydrolase fami...
Sobic.010G246700		+		+	XTH25, XTR3	xyloglucan endotransglucosylase/hydrolase 25
Sobic.003G442500		-		-	ATCSLG3, CSLG3	cellulose synthase like G3
Sobic.004G237800		-		-	GAUT8, QUA1	Nucleotide-diphospho-sugar transferases superf...
Sobic.003G436800		-				Peroxidase superfamily protein
Sobic.009G216100		-				Pectin lyase-like superfamily protein
Sobic.006G191700	+		+		ATEXP13, ATEXPA13, ATHEXP ALPHA 1.22, EXP13, E...	expansin A13
Sobic.002G324100			-		XTH8	xyloglucan endotransglucosylase/hydrolase 8
Sobic.006G205600	-		-		MERI-5, MERI5B, SEN4, XTH24	xyloglucan endotransglucosylase/hydrolase 24
Sobic.010G008600			-		ATCSLD3, CSLD3, KJK	cellulose synthase-like D3
Sobic.010G246400	-		-		TCH4, XTH22	Xyloglucan endotransglucosylase/hydrolase fami...
Sobic.003G232600	+					Pectin lyase-like superfamily protein
Sobic.003G127100	-					Peroxidase superfamily protein

Table S10: GO term "cell wall organization" highly variable genes (root). List of genes found as highly variable for either genotypes in one of the following categories: Preflowering during the drought period, preflowering during the recovery period, or postflowering. The direction of the change is marked by a + (overexpressed in treatment) or - (underexpressed in treatment). The last two columns correspond to the closest homolog in arabidopsis and its description.

Sobic.001G012300	+	+		+	+	+	CYP79B2	cytochrome P450, family 79, subfamily B, polyp...
Sobic.004G028700		+				+		Pectin lyase-like superfamily protein
Sobic.005G169500	+		+	+		+	GAUT6, GAUT4	galacturonosyltransferase 6,galacturonosyltran...
Sobic.007G094900	+	+	+	+	+	+	TCH4, XTH22	Xyloglucan endotransglucosylase/hydrolase fami...
Sobic.009G032800	+	+			+	+	RCI3, RCI3A	Peroxidase superfamily protein
Sobic.009G216100		+			+	+		Pectin lyase-like superfamily protein
Sobic.001G045800	-	+		-		-	ADPG2, PGAZAT	polygalacturonase abscission zone A. thaliana
Sobic.001G086000						-	COB	COBRA-like extracellular glycosyl-phosphatidyl...
Sobic.001G189000		+				-		Peroxidase superfamily protein
Sobic.001G189200						-		Peroxidase superfamily protein
Sobic.001G224300			-			-	CESA4, IRX5, NWS2	cellulose synthase A4
Sobic.001G237900			-	-		-	ATEXP11, AT-EXPA11, ATHEXP ALPHA 1.14, EXP11, E...	expansin 11
Sobic.001G238400						-	ATEXP11, AT-EXPA11, ATHEXP ALPHA 1.14, EXP11, E...	expansin 11
Sobic.001G314000		+				-	RCI3, RCI3A	Peroxidase superfamily protein
Sobic.001G345300						-		Plant invertase/pectin methylesterase inhibito...
Sobic.001G499900		+				-	ATEXP11, AT-EXPA11, ATHEXP ALPHA 1.14, EXP11, E...	expansin 11
Sobic.002G003200		+	-			-		Peroxidase superfamily protein
Sobic.002G003700		+				-	RCI3, RCI3A	Peroxidase superfamily protein
Sobic.002G205500		+	-			-	ATCESA7, CESA7, IRX3, MUR10	Cellulose synthase family protein
Sobic.002G333900	-	-	-	-		-	ATCSLD3, CSLD3, KJK	cellulose synthase-like D3
Sobic.002G334000			-			-	ATCSLD5, CSLD5, SOS6	cellulose synthase-like D5
Sobic.002G334100		+	-	-		-	ATCSLD5, CSLD5, SOS6	cellulose synthase-like D5
Sobic.002G334200	-	+	-	-		-	ATCSLD5, CSLD5, SOS6	cellulose synthase-like D5
Sobic.002G391300		+	-			-		Peroxidase superfamily protein
Sobic.002G391400	-		-			-		Peroxidase superfamily protein
Sobic.002G391900	-	+	-			-		Peroxidase superfamily protein
Sobic.002G392000		+	-	-		-		Peroxidase superfamily protein
Sobic.002G392100	-	+	-	-		-		Peroxidase superfamily protein
Sobic.003G050300	-			-		-		Peroxidase superfamily protein
Sobic.003G140600	-	+	-	-		-		Peroxidase superfamily protein
Sobic.003G140700	-	+	-	-		-		Peroxidase superfamily protein
Sobic.003G148300	-	+		-	+	-	ATPMEPCRB	Plant invertase/pectin methylesterase inhibito...
Sobic.003G152000	-		-			-		Peroxidase superfamily protein
Sobic.003G152200	-		-	-		-		Peroxidase superfamily protein
Sobic.003G167300	-		-			-		Peroxidase superfamily protein
Sobic.003G232600		+				-		Pectin lyase-like superfamily protein
Sobic.003G292100					-	-		Pectin lyase-like superfamily protein
Sobic.003G296400			-			-	ATCESA8, CESA8, IRX1, LEW2	cellulose synthase family protein
Sobic.003G338801			-			-	AT-EXP1, AT-EXP1, ATEXPA1, ATHEXP ALPHA 1.2, EX...	expansin A1
Sobic.004G013500		+	-			-	AtUGP2, UGP2	UDP-glucose pyrophosphorylase 2
Sobic.004G113900						-		Pectin lyase-like superfamily protein
Sobic.004G273900	-		-	-		-	RCI3, RCI3A	Peroxidase superfamily protein
Sobic.005G051500	-			-		-		Peroxidase superfamily protein

Continued on next page

Table S10: GO term “cell wall organization” highly variable genes (root). List of genes found as highly variable for either genotypes in one of the following categories: Preflowering during the drought period, preflowering during the recovery period, or postflowering. The direction of the change is marked by a + (overexpressed in treatment) or - (underexpressed in treatment). The last two columns correspond to the closest homolog in arabidopsis and its description.

Sobic.005G140001		+	-			-	EXGT-A4, XTH5	xyloglucan endotransglucosylase/hydrolase 5
Sobic.006G031900		+	-			-	ATEXP11, AT-EXPA11, ATHEXP ALPHA 1.14, EXP11, E...	expansin 11
Sobic.006G116000			-			-	SVL1	SHV3-like 1
Sobic.006G205500					+	-	XTH25, XTR3	xyloglucan endotransglucosylase/hydrolase 25
Sobic.006G205700		+	-			-	XTH16	xyloglucan endotransglucosylase/hydrolase 16
Sobic.006G217900	-		-	-		-	FLS2	Leucine-rich receptor-like protein kinase fami...
Sobic.006G224500			-			-		Peroxidase superfamily protein
Sobic.006G273300						-		Uncharacterised protein family (UPF0497)
Sobic.006G277500	-		-	-		-		Peroxidase superfamily protein
Sobic.006G277550				-	+	-		Peroxidase superfamily protein
Sobic.006G277600						-		Peroxidase superfamily protein
Sobic.006G277700		+				-		Peroxidase superfamily protein
Sobic.006G277800	-		-			-		Peroxidase superfamily protein
Sobic.007G014200	-		-	-		-		Peroxidase superfamily protein
Sobic.007G018000	-			-		-	ATEXP25, AT-EXPA25, ATHEXP ALPHA 1.18, EXP25, E...	expansin A25
Sobic.007G086300			-			-	TCH4, XTH22	Xyloglucan endotransglucosylase/hydrolase fami...
Sobic.008G125700			-			-	ATCSLD5, CSLD5, SOS6	cellulose synthase-like D5
Sobic.009G033500	-	-	-	-		-		Peroxidase superfamily protein
Sobic.009G186500		+	-			-	RCI3, RCI3A	Peroxidase superfamily protein
Sobic.009G203200						-		Pectin lyase-like superfamily protein
Sobic.009G250800						-		Pectin lyase-like superfamily protein
Sobic.010G128700		+	-			-		Peroxidase superfamily protein
Sobic.010G194500			-			-	ATEXP20, AT-EXPA20, ATHEXP ALPHA 1.23, EXP20, E...	expansin A20
Sobic.K026900	-	+	-	-		-		Peroxidase superfamily protein
Sobic.010G246600			+				TCH4, XTH22	Xyloglucan endotransglucosylase/hydrolase fami...
Sobic.001G238200	-		-	-			ATEXP11, AT-EXPA11, ATHEXP ALPHA 1.14, EXP11, E...	expansin 11
Sobic.002G302000		+	-				XTH32	xyloglucan endotransglucosylase/hydrolase 32
Sobic.002G368300			-				COBL4, IRX6	COBRA-like extracellular glycosyl-phosphatidyl...
Sobic.002G391200			-					Peroxidase superfamily protein
Sobic.002G416000			-				ATPME2, PME2	pectin methylesterase 2
Sobic.002G420100			-				GAUT7, LGT7	galacturonosyltransferase 7
Sobic.003G148400		+	-				ATPME2, PME2	pectin methylesterase 2
Sobic.003G223100			-		-			Pectin lyase-like superfamily protein
Sobic.006G106900	-	+	-					Uncharacterised protein family (UPF0497)
Sobic.007G075600			-					Pectin lyase-like superfamily protein
Sobic.007G146200	-		-	-				Plant invertase/pectin methylesterase inhibito...
Sobic.008G114700			-				RCI3A, RCI3	Peroxidase superfamily protein
Sobic.009G055300		+	-		+		RCI3, RCI3A	Peroxidase superfamily protein
Sobic.001G283400		+			+		ATCSLD3, CSLD3, KJK	cellulose synthase-like D3
Sobic.002G370300					+		QRT1	Pectin lyase-like superfamily protein
Sobic.003G050100	+				+			Pectin lyase-like superfamily protein
Sobic.003G141800					+			Pectin lyase-like superfamily protein
Sobic.003G153100	+	+		+	+			Pectin lyase-like superfamily protein
Sobic.003G437400					+			Peroxidase superfamily protein
Sobic.004G315000		+			+		TBR	Plant protein of unknown function (DUF828)

Continued on next page

Table S10: GO term "cell wall organization" highly variable genes (root). List of genes found as highly variable for either genotypes in one of the following categories: Preflowering during the drought period, preflowering during the recovery period, or postflowering. The direction of the change is marked by a + (overexpressed in treatment) or - (underexpressed in treatment). The last two columns correspond to the closest homolog in arabidopsis and its description.

Sobic.007G090436		+		+	TCH4, XTH22	Xyloglucan endotransglucosylase/hydrolase fami...
Sobic.009G111000	-		-	+	ATPMEPCRB	Plant invertase/pectin methylesterase inhibito...
Sobic.001G085200		+			PDCB3	plasmodesmata callose-binding protein 3
Sobic.001G238000		+			ATEXP11, AT-EXPA11, ATHEXP ALPHA 1.14, EXP11, E...	expansin 11
Sobic.001G336700		+			COBL4, IRX6	COBRA-like extracellular glycosyl-phosphatidyl...
Sobic.001G360400	-	+				Peroxidase superfamily protein
Sobic.001G525000		+				Pectin lyase-like superfamily protein
Sobic.003G096300		+	+		HDG1, HD-GL2-1	homeodomain GLABROUS 1
Sobic.003G153200	+	+	+			Pectin lyase-like superfamily protein
Sobic.003G436800	-	+	-			Peroxidase superfamily protein
Sobic.004G126700		+			AtXTH12, XTH12	xyloglucan endotransglucosylase/hydrolase 12
Sobic.004G231300		+			OSU1, QUA2, TSD2	S-adenosyl-L-methionine-dependent methyltransf...
Sobic.004G313900	+	+			ATHDG11, EDT1, HDG11	homeodomain GLABROUS 11
Sobic.007G090460		+			AtXTH13, XTH13	xyloglucan endotransglucosylase/hydrolase 13
Sobic.009G033400		+			ATPA2, PA2	peroxidase 2
Sobic.009G243500		+				Pectin lyase-like superfamily protein
Sobic.010G232500		+			RCI3, RCI3A	Peroxidase superfamily protein
Sobic.001G284600		-			ATXTH27, EXGT-A3, XTH27	endoxyloglucan transferase A3
Sobic.001G309000	-	-			ATXTR8, XTH31, XTR8	xyloglucan endo-transglycosylase-related 8
Sobic.002G237900	-	-	-		ATCSLE1, CSLE1	cellulose synthase like E1
Sobic.002G427400	-	-	-		ATSEB1, COBL7, SEB1	COBRA-like protein-7 precursor
Sobic.003G178000		-	-			Pectin lyase-like superfamily protein
Sobic.009G033300	-	-	-			Peroxidase superfamily protein
Sobic.001G499800	+		+		ATEXP11, AT-EXPA11, ATHEXP ALPHA 1.14, EXP11, E...	expansin 11
Sobic.004G238801			+		AT-EXP1, AT-EXP1, ATEXPA1, ATHEXP ALPHA 1.2, EX...	expansin A1
Sobic.006G172000	+		+			Pectin lyase-like superfamily protein
Sobic.001G238300	-		-		ATEXP11, AT-EXPA11, ATHEXP ALPHA 1.14, EXP11, E...	expansin 11
Sobic.003G321200	-		-		ATPMEPCRA, PMEPCRA	methylesterase PCR A
Sobic.004G237800			-		GAUT8, QUA1	Nucleotide-diphospho-sugar transferases superf...
Sobic.007G085600	-		-		XTH15, XTR7	xyloglucan endotransglucosylase/hydrolase 15
Sobic.009G055100	-		-		RCI3, RCI3A	Peroxidase superfamily protein
Sobic.010G008600			-		ATCSLD3, CSLD3, KJK	cellulose synthase-like D3
Sobic.010G017600			-			Plant invertase/pectin methylesterase inhibito...
Sobic.003G320800	-				RCI3, RCI3A	Peroxidase superfamily protein
Sobic.003G442500	-				ATCSLG3, CSLG3	cellulose synthase like G3
